# Nowcasting COVID‐19 incidence indicators during the Italian first outbreak

**DOI:** 10.1002/sim.9004

**Published:** 2021-05-06

**Authors:** Pierfrancesco Alaimo Di Loro, Fabio Divino, Alessio Farcomeni, Giovanna Jona Lasinio, Gianfranco Lovison, Antonello Maruotti, Marco Mingione

**Affiliations:** ^1^ Department of Statistical Sciences University of Rome “La Sapienza” Rome Italy; ^2^ Department of Bio‐Sciences University of Molise Campobasso Italy; ^3^ Department of Economics and Finance University of Rome “Tor Vergata” Rome Italy; ^4^ Department of Economics, Management and Statistics University of Palermo Palermo Italy; ^5^ Department of Epidemiology and Public Health Swiss TPH Basel Basel Switzerland; ^6^ Department of GEPLI Libera Universitá Maria Ss Assunta Rome Italy; ^7^ Department of Mathematics University of Bergen Bergen Norway; ^8^ IAC ‐ CNR Institute of Applied Computing “M. Picone” Rome Italy

**Keywords:** COVID‐19, growth curves, Richards' equation, SARS‐CoV‐2

## Abstract

A novel parametric regression model is proposed to fit incidence data typically collected during epidemics. The proposal is motivated by real‐time monitoring and short‐term forecasting of the main epidemiological indicators within the first outbreak of COVID‐19 in Italy. Accurate short‐term predictions, including the potential effect of exogenous or external variables are provided. This ensures to accurately predict important characteristics of the epidemic (e.g., peak time and height), allowing for a better allocation of health resources over time. Parameter estimation is carried out in a maximum likelihood framework. All computational details required to reproduce the approach and replicate the results are provided.

## 1 INTRODUCTION

Italy has been the first European country to be severely hit by the first epidemic wave due to the spread of the SARS‐CoV‐2 virus. COVID‐19 syndrome emerged in northern Italy in February 2020, with a basic reproduction number *R*_0_ between 2.5 and 4.^1^ In its most severe form, COVID‐19 has two challenging characteristics:^2^ it is highly infectious and, despite having a benign course in the vast majority of patients, it requires hospital admission and even intensive care for about 10% of those infected.^3^ During the outbreak, it was crucial to set up appropriate data collection and modeling systems quickly. Both were necessary for monitoring, evaluation of policy interventions, and prediction.

Generally speaking, the nature of epidemics' spread has nearly always followed the same scenario: first, the growth in the number of infected people is (close to) exponential; in a second moment, this growth gradually but consistently slows down as an effect, for instance, of various containment measures. This pattern can cyclically recur until the outbreak is tamed.

So far, in order to explain the spread of epidemics and predict their consequences, a number of mathematical and statistical models of different complexity levels have been used. The starting point is often the Verhulst logistic equation,^4^ which can easily capture both the exponential increase in the number of infected people at the initial stage of the epidemic development, and the tendency towards a constant value by its ending. In more complex models, people are divided into different groups: (S) the susceptible class, namely those individuals who are capable of contracting the disease and becoming infected; (I) the infected class, namely those individuals who are capable of transmitting the disease to others; (R) the removed class, namely infected individuals who are deceased or have recovered, who are either permanently immune or isolated. This group of mathematical models are called SIR (or compartmental) models.^5^ References include,^6^, ^7^, ^8^, ^9^ and several more. However, whilst being potentially very appropriate to model the dynamics underlying any epidemic, SIR‐based models rely on accurate initial estimates of several quantities governing its spreading mechanism (which are unknown). Poor data input on key features of the pandemic can heavily bias these estimates, jeopardizing the reliability of any theory‐based forecasting effort. SIR models are microsimulation models and we believe that, generally speaking, they should be used mostly for “scenario evaluation” rather than predicting future outcomes. Indeed, they rely on several speculations and strict theoretical assumptions, not necessarily met by the analyzed data and, especially during the first stage of the outbreak, failed in predicting various COVID‐19 related outcomes.^10^ Such specifics lead the choice of coefficients in the equations defining the SIR model and define its initial conditions. It is well known that even a slight change in those can lead to large differences in the final results. For instance, at the beginning of the epidemic, early data providing estimates for case fatality rate, infection fatality rate, basic reproductive number, and other key numbers that are essential for the modeling, are often inflated and may cause potentially large overestimation of the epidemic severity. Similar criticism to using compartmental modeling for nowcasting can also be found in Reference 11, and references therein. Hence, we have preferred to follow an alternative approach, which involved direct modeling of the observed counts.^12^ This encompasses the use of phenomenological models without detailed mechanistic foundations, but which have the advantage of allowing simple calibrations to the empirical reported data. Such approaches are particularly suitable when substantial uncertainty tarnishes the epidemiology of an infectious disease, including the potential contribution of multiple transmission pathways. In these situations, phenomenological models provide a starting point for obtaining early estimates of the transmission potential and short‐term forecasts of the epidemic evolution.^13^

We propose a parametric regression model for the modeling of *incidence indicators* (defined in Section 2.1) based on the use of the Richards' curve (a generalized logistic function) in place of the widely used exponential or polynomial trends. Furthermore, we replace the generally entrenched Gaussian assumption for the distribution of log‐counts^14^, ^15^ by the more appropriate Poisson or Negative Binomial distributions for counts. In this way we avoid the implausible assumptions stemming from the more common alternatives: the former allows the underlying counts to potentially grow indefinitely; the latter neglects the proper specification of dependence between mean and variance under the log‐normal distribution. We further propose different ways of including the effect of exogenous information on the response function of counts, in an extended generalized linear model framework. These models have been implemented during the outbreak with the aim of modeling the medium to long term evolution of the epidemic wave. The use of logistic‐based curves is also widely discussed in the literature.^16^, ^17^, ^18^ Logistic growth curves can be seen as a flexible formulation for approximating a large variety of growth phenomena, especially in biology and in epidemiology.^19^, ^20^, ^21^, ^22^, ^23^ In particular, highly flexible parametric models such as Gompertz curves and the unified Richards' family^24^ have been proposed in the study of organisms' growth, for a review see Reference 25.

The article is organized as follows: Section 2 gives a detailed description of the Italian situation and provides a brief account of the Italian public data made available daily, with some remarks on limitations and flaws in the data collection process; Section 3 contains a description of our approach to modeling incidence indicators, including remarks on how to obtain standard errors for parameters and predictions performances; Section 4 illustrates results of our approach applied to the incidence indicators recorded during first wave of the Italian outbreak of COVID‐19. Finally, results are discussed and commented along with some concluding remarks in Section 5.

The methods discussed in this article have also been implemented in a Shiny app, publicly available at https://statgroup19.shinyapps.io/StatGroup19‐Eng/.

## 2 AVAILABLE DATA AND THEIR LIMITATIONS

The Italian Civil Protection Department (CPD), starting from February 24th, 2020, has been gathering data at the regional level every day and making these public in a GitHub repository. During most of the Italian epidemic, data were commented by the department head in an official press release at about 6 pm. The daily updated data are currently stored at https://github.com/pcm‐dpc/COVID‐19. For public health service purposes, Italy is divided into 21 regions. There are 19 administrative regions, plus two autonomous provinces (Trento and Bolzano) that form the administrative region of Trentino‐Alto‐Adige. In the sequel, we focus on modeling the indicators aggregated at the national level. Nevertheless, the supplementary material contains graphical and quantitative performances of our model on the 21 single regions.

### 2.1 Incidence and prevalence indicators: different mathematical features

The epidemiological data provided by CPD can be distinguished into two basic types:

1. incidence indicators (flows)
2. prevalence indicators (stocks)

#### 2.1.1 Incidence indicators

Incidence indicators measure the number of individuals with a particular condition, related with the epidemic, recorded during a given period. They can be referred to different time periods; in particular, in the CPD dataset, *daily incidence counts* are available for the following indicators:

- positives, which are subclassified into two subconditions:
  – hospitalized (either in regular wards or in ICU)
  – isolated‐at‐home
- deceased
- recovered/discharged

**FIGURE 1:**
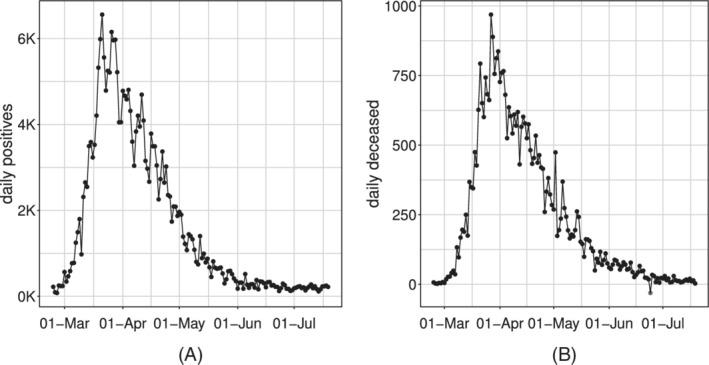
Time series of the Italian daily incidence indicators: *daily positives*, A and *daily deceased*, B

These indicators can be considered, by analogy with the terminology used in econometrics, as *flow data*, quantifying the daily input (positives) and output (deceased and recovered/discharged) of the system. The time series of *daily positives* and *daily deceased* aggregated at the national level are shown in Figure 1. From the viewpoint of the following modeling effort, one important feature of these indicators is that they can be referred to longer time intervals, simply cumulating them over time. The most interesting *cumulative incidence indicators* are those referring to the whole history of the pandemic, computed from a conventional date of “beginning of the pandemic” (typically, the day the systematic recording of daily positives began) to the current day:

- cumulative positives
- cumulative deceased
- cumulative recovered/discharged

In particular, given *Y*_0_ = 0, we can build the whole series of cumulative counts conditionally on the value of the cumulative indicator at time (*t* − 1), and the incidence indicators at time *t*, for each *t* = 1, … , *T*:

$$Y_{t}^{c}=Y_{t-1}^{c}+I_{t},$$

where $Y_{t}^{c}$ represents the cumulative indicator and *I*_*t*_ represents the inputs in the system, for example: cumulative positives at time *t* are the cumulative positives at time (*t* − 1) plus the daily positives at day *t*.

**FIGURE 2:**
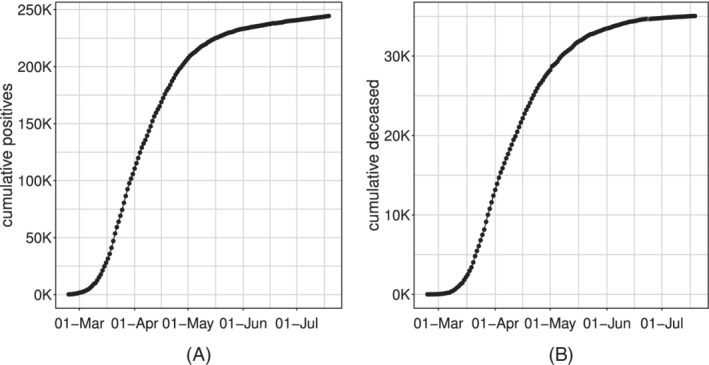
Time series of the Italian cumulative incidence indicators: *cumulative positives*, A and *cumulative deceased*, B

By their nature of cumulative counts, these data series are necessarily monotonically nondecreasing (see Figure 2 for the *positives* and *deceased* example).

#### 2.1.2 Prevalence indicators

Prevalence indicators measure the number of individuals with a particular condition, related with the epidemic, at a given instant in time (or at a given short interval of time, eg, a day). They are typically obtained from simple algebra from other indicators; in particular, in the CPD dataset, the following indicators are available daily:

- current positives;
- current *intensive care units* (ICU) occupancy.

**FIGURE 3:**
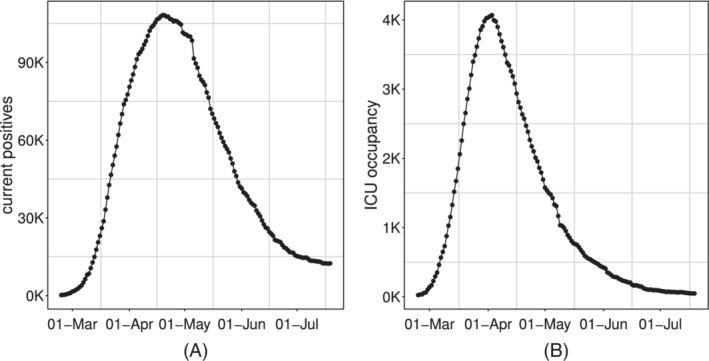
Time series of Italian daily prevalence indicators: *current positive*, A and *ICU occupancy*, B

These indicators result from the balance between total inputs and outputs of the system, for example: current positives are the difference between cumulative positives and cumulative deceased plus recovered/discharged. Again, by analogy with the terminology used in econometrics, they can be considered as *stock data*. In particular, given *Y*_0_ = 0, we can build the whole series conditionally on the value of the prevalence indicator at time (*t* − 1), and the incidence indicators at time *t*, for each *t* = 1, … , *T*:

$$Y_{t}^{p}=Y_{t-1}^{p}+I_{t}-O_{t},$$

where $Y_{t}^{p}$ represents the prevalence indicator, *I*_*t*_ represents the inputs in the system and *O*_*t*_ represents the outputs, for example: current positives at time *t* are the current positives at time (*t* − 1) plus the daily positives at day *t* and minus the sum of deceased and discharged recovered at day *t*. However, given the different delay in reporting the various information by the regional agencies, there exists a relevant temporal misalignment among all the quantities reported at the daily scale. Therefore, the simultaneous consideration of all these flows may be significantly flawed and we rather prefer modeling the indicators individually. Two important features of these indicators are that:

1. given their *stock* nature, they cannot be aggregated (eg,: it does not make sense to compute “cumulative current positives”);
2. by their own nature, these indicators are not monotone, since they can increase or decrease as a result of different trends of the component series. Typically, we expect the series of current positives and ICU occupancy to increase in the rising phase of an epidemic, reach a peak and then decrease to a lower asymptote (see Figure 3), although more complex patterns due to resurgence of the epidemic are also plausible.

Prevalence indicators are characterized by a strong and tangled dependence structure which is cumbersome to simplify into a manageable and useful statistical model on the short run.

For this reason, the focus of this work concerns only incidence indicators. Our model proposal, from a strictly mathematical point of view, could potentially applied also on prevalence indicators. However, from the statistical point of view, the modeling assumptions which are assumed to hold (with good approximation) considering the incidence indicators, are likely to be strongly violated by prevalence indicators and the resulting outcome cannot be considered reliable. A brief discussion about some possible approaches for the analysis of prevalence indicators is given in Section 5.

### 2.2 Data issues

COVID‐19 public Italian data present several issues that severely affect their quality. The information has been gathered and reported at a regional level, and each regional healthcare organization has a different transmission and data collection system*.

Measurement errors, and errors in data entry, are expected to be often present. Delays in reporting has been, sometimes, substantial. Some patients were transferred (eg, from Lombardia to Puglia, and even to Germany) without notification, and they were counted as hospital patients of the receiving region (or not at all when sent abroad) and positive cases of the region of residence. Most importantly, counts were updated on the notification day rather than aligned to a more appropriate date. For example, death is counted on the day of the reporting, not on the day of the outcome, which could be even weeks before. Positive status is also counted on the day that test results are received, with swabs being processed from one day to weeks after symptoms' onset. No distinction between actively symptomatic and asymptomatic patients was made.

Swabs and positive cases are not time‐aligned. For example, in countries like Singapore (https://www.moh.gov.sg/covid‐19), daily data include information on total swabs tested, total unique persons swabbed as well as total swabs per 1 000 000 total population and total unique persons swabbed per 1 000 000 total population. In Italy, up to April 19th, 2020, only the total number of daily swabs is available, and no linkage between swabs and tested individuals was kept in the data repository. Hence, it is impossible to make statistically sound use of swabs' count to model the whole first pandemic wave.

Finally, it is crucial to recall that people diagnosed with COVID‐19 disease are only a small fraction of the people infected by the virus. Moreover, since the tracking was highly symptoms driven, especially in the first phase of the outbreak, the detected number of positives cases can provide only a partial estimate of the *true* incidence of COVID‐19 in the Italian population. Eventually, we expect this detected fraction to vary wildly over space and time.

In our opinion, the most reliable indicator is the count of ICU occupancy. The reason is that the Italian Society for Emergency Care issued national guidelines (that did not change substantially during the epidemic) for testing patients with a suspected infection by SARS‐CoV‐2, who also had top priority for swab access and reporting; and ICU admissions can be expected to depend on the proportion of infected population susceptible to severe infection, rather than on the regional strategy for testing and contact tracing. However, while probably reliable, this indicator also presents some drawbacks. First of all, it provides only a partial snapshot of the epidemic's current stage, which concerns the most severe cases of the disease. The latter is a critical issue, especially in the COVID‐19 case, which is known to present severe symptoms only in a small percentage of the currently affected individuals. Second, this snapshot is affected by a constant delay (ie, the time between catching the disease and manifesting severe symptoms). As mentioned in Section 2.1.2, its daily variation is obtained as a combination of new incoming patients (+) and the deceased or recovered ones (‐), whose effects blend and are hard to disentangle. As a consequence, *incidence indicators*, such as *daily positives* and *daily deceased*, while being measured with some error and even more delay in the case of deaths, still represent the critical indicators for timely and appropriate monitoring of the pandemic.

## 3 MODEL SPECIFICATION

The time series of any of the observed indicators, denoted by $z={\left\{z_{t}\right\}}_{t=t_{0}}^{T}$, is modeled separately and considered as the realization of the stochastic process $Z={\left\{Z_{t}\right\}}_{t=t_{0}}^{T}$. The idea behind this article is to model any of the mentioned indicators through a Generalized Model with a response function $𝔼[Z_{t}]=\mu (t)=g^{-1}\left(t;\theta \right)$, where *g*(·) is a known link function and θ is a parameter vector, that is appropriate for the specific mathematical features of the epidemic process. This must be coupled with a response distribution $f(Z_{t};\theta )$ coherent with the domain of such indicators, which are counts and therefore Natural numbers.

### 3.1 Response function for incidence indicators

**FIGURE 4:**
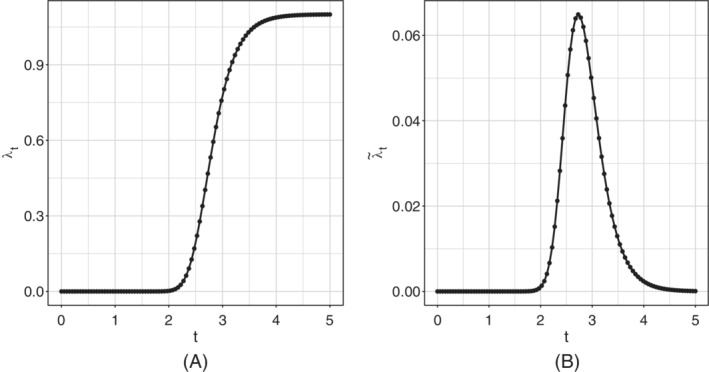
Example of Richards' curve, A and derivative of the Richards' curve, B

Let us denote by ${\left\{y_{t}^{c}\right\}}_{t=0}^{T}$ the time‐series of cumulative incidence indicators since the start of the epidemic (*t*_0_ = 0, first day of systematic data recording). Visual inspection of these indicators in Figure 2 suggests that their expected values follow a logistic‐type growth curve. Different example of logistic curves have been proposed in the literature, all representing solutions to specific differential equations that model the spread of epidemics.^26^, ^27^, ^28^ Differently from the more standard exponential models, these are able to describe the slowdown of the outbreak associated with a decaying transmission rate just after the number of cases approaches its inflection point. They have been already widely used to describe the evolution of the COVID‐19 pandemic in different states during its early to medium stage.^29^, ^30^ Here, for all the incidence indicators, we consider the *Generalized Logistic Function*, also known as *Richards'* curve (see Figure 4 as an example), as response function for the mean of the process.^31^ This curve was widely used to describe various biological processes,^32^ but has been recently adapted also in epidemiology for real‐time prediction of outbreak of diseases.^33^, ^34^, ^35^ The specialty of the Richards' curve lies in its ability to describe a great variety of growing processes, endowed with strong flexibility, that includes as special cases the standard logistic growth curve,^36^ the Gompertz growth curve^37^ and others. It can be expressed in different forms.^38^, ^39^, ^40^, ^41^ One of its most general formulation depends on the vector of five parameters $\gamma^{⊤}=[b,r,h,p,s]$ and can be expressed as:

(1) $$𝔼[Y_{t}^{c}]=g^{-1}(t;\gamma )=\lambda_{\gamma }(t)=b+\frac{r}{{\left(1+10^{h(p-t)}\right)}^{s}}.$$

$b\in {\mathbb{R}}^{+}$ represents a lower asymptote and *r* > 0 is the distance between the upper and the lower asymptote, hence *b* + *r* would be the final epidemic size; *h* is known as the *hill*, and represents the infection/growth rate; p∈ℝ represents a lag‐phase of the trajectory and determines the peak position (it tells when the curve growth speed slows down); s∈ℝ is an asymmetry parameter regulating differences in the behavior of the ascending and descending phase of the outbreak. In our context, since cumulative incidences are always monotone increasing indicators, it is reasonable to assume *h*, *s* > 0 †.

An extensive review of the Richards' curve and other logistic growth models, together with discussion on the proper interpretation of the parameters, is given in Reference 24.

An *Extended Generalized Linear Model* with (1) as response function seems to be a natural choice for modeling time series of *cumulative counts*, whose monotonically nondecreasing average behaves as the *Richards'* curve. Unfortunately, there is a significant drawback to this choice. As it will be better clarified in Section 3.2, a very useful working assumption would be that all these counts were stochastically independent, given their mean function $\lambda_{\gamma }(t)$. However, we cannot consider this assumption as realistic in the case of cumulative counts, since the constraint on the domain of definition on subsequent counts (ie, $y_{t}^{c}\geq y_{\tau }^{c},\forall \tau <t$) is not guaranteed to be satisfied. On the other hand, the stochastic independence assumption sounds more reasonable, albeit not necessarily true, for the *daily incidence counts* ${\left\{y_{t}\right\}}_{t=1}^{T}$, that is, the addenda of the cumulative counts excluding the starting point *y*_0_, which can be defined as:

$$y_{t}^{c}=\overset{t}{\underset{\tau =0}{\sum }}y_{\tau }\;\Rightarrow \;y_{t}=y_{t}^{c}-y_{t-1}^{c},\;t=1,\ldots ,T,$$

where *y*_0_ = 0 by definition.

Using Equation (1), and exploiting the additive properties of the expected value, we have:

$$\begin{array}{llll} \tilde{\mu }(t)=𝔼[Y_{t}] & =𝔼[Y_{t}^{c}]-𝔼[Y_{t-1}^{c}]=\lambda_{\gamma }(t)-\lambda_{\gamma }(t-1)= & & \\ & =r\cdot \left[{\left(1+10^{h(p-t)}\right)}^{-s}-{\left(1+10^{h[p-(t-1)]}\right)}^{-s}\right]={\tilde{\lambda }}_{\gamma }(t) & & \end{array}$$

which, in particular, does not depend on the baseline *b*. Therefore, we shall adopt an extended Generalized Model with response function given by the first differences of the Richards' curve $\tilde{\lambda_{\gamma }}={\left\{{\tilde{\lambda }}_{\gamma }(t)\right\}}_{t=1}^{T}$ to model the daily expected values $\mu ={\left\{\mu (t)\right\}}_{t=1}^{T}$ of the observed incidence counts $y={\left\{y_{t}\right\}}_{t=1}^{T}$ (see example in Figure 4).

In addition, we may also consider adding a kink effect/baseline α to the first differences ${\tilde{\lambda }}_{\gamma }(\cdot )$, which is to say assuming the following functional form for the mean of the daily counts:

(2) $${\tilde{\mu }}_{\theta }(t)=\alpha +{\tilde{\lambda }}_{\gamma }(t),\;\alpha \geq 0,$$

where θ=(α,γ). This would correspond to the following mean function for the cumulative counts:

$$\mu_{\theta }(t)=\alpha \cdot (t-1)+\lambda_{\gamma }(t).$$

In practice, the parameter α includes the possibility of having a strictly positive baseline rate, which can be interpreted as the *endemic steady state incidence rate*. This is in line with the current perspective that SARS‐CoV‐2 might not be completely eradicated within the next few years.^42^ On the other hand, the first differences of the Richards' curve ${\tilde{\lambda }}_{\gamma }(t)$ are (by construction) forced to decrease asymptotically to the value of 0. However, this asymptotic result is not necessarily observed in real data. In particular, Figure 1 highlights that both time‐series do not attain the 0 value, but settle to a low, constant level. This situation may, potentially, continue indefinitely: new cases will be found as long as people will be tested. Consequently, the model without a baseline lacks the ability to catch this tail and, because of the curve parametric form, this may indirectly affect the fit on the whole series.

In the first instance, one solution would be to fit the model, including the kink effect α. Afterward, if it is estimated not to be sensibly different from 0, the model without α can be fitted again to stabilize the estimation procedure and decrease the uncertainty on the other parameters.

### 3.2 Response distribution for incidence indicators

Before introducing the distributions for the daily incidence counts, we must make some assumptions about the time dependence structure. In particular, we assume that given the mean function ${\tilde{\mu }}_{\theta }(t)$, the daily incidence counts *Y*_*t*_ are stochastically independent from the previous cumulative counts: $Y_{t}⊥Y_{\tau }^{c}\;\forall \;\tau <t$. We denote this hypothesis of independence by *HI*. We also assume the value of the first cumulative count $Y_{0}^{c}=y_{0}^{c}$ to be known and fixed. Exploiting *HI*, we can express the joint density of all the subsequent cumulative counts conditional on $Y_{0}^{c}=y_{0}^{c}$ as the product of the univariate densities of the corresponding daily counts ${\left\{Y_{t}\right\}}_{t=1}^{T}$. The equivalence follows from the following conditional argument:

$$\begin{array}{llll} f_{Y_{1}^{c},\ldots ,Y_{T}^{c}}(y_{1}^{c},\ldots ,y_{T}^{c}|y_{0}^{c};\theta ) & =\overset{T}{\underset{t=1}{\prod }}f_{Y_{t}^{c}}(y_{t}^{c}|y_{0}^{c},\ldots ,y_{t-1}^{c};\theta )=\overset{T}{\underset{t=1}{\prod }}f_{Y_{t}^{c}}(y_{t}+y_{t-1}^{c}|y_{0}^{c},\ldots ,y_{t-1}^{c};\theta )= & & \\ & =\overset{T}{\underset{t=1}{\prod }}f_{Y_{t}}(y_{t}|y_{0}^{c},\ldots ,y_{t-1}^{c};\theta )\overset{\text{HI}}{=}\overset{T}{\underset{t=1}{\prod }}f_{Y_{t}}(y_{t}|\theta ), & & \end{array}$$

where the second identity is justified in the light of $Y_{t}^{c}=Y_{t}+Y_{t-1}^{c},\;t=1,\ldots ,T$, which is true by definition. From a practical point of view, this also implies a first‐order Markov property for the cumulative counts:

$$Y_{t}^{c}|Y_{t-1}^{c}⊥Y_{1}^{c},\ldots ,Y_{t-2}^{c},\;t=1,\ldots ,T$$

and mutual independence between the daily counts:

$$Y_{t}⊥Y_{\tau },\;\forall \;t,\tau ,t\neq \tau .$$

We remark that although these independence structure is just an approximation in the present case, this kind of approach has provided valid inference for all the available Italian incidence indicators.

For communication purposes, it can be of interest to report the results of analyses and predictions in terms of cumulative, rather than daily, incidence indicators. Clearly, it is possible to model and predict the daily incidence indicators and, from these estimates and predictions, obtain the relevant cumulative incidence indicators.

#### 3.2.1 Poisson distribution

Let us assume that the vector of daily incidence counts, $y=\left\{y_{1},\ldots ,y_{t}\right\}$, is composed of independent Poisson realizations with expected value ${\tilde{\mu }}_{\theta }(t)$:

$$Y_{t}|\theta ∼\text{Pois}({\tilde{\mu }}_{\theta }(t)),\;t=1,\ldots ,T.$$

Hence, the likelihood can be written as:

$$\begin{array}{ll} ℒ(\theta |y) & =\overset{T}{\underset{t=1}{\prod }}\text{Pois}(y_{t}|{\tilde{\mu }}_{\theta }(t))\propto \\ & \propto {\tilde{\mu }}_{\theta }{\left(t\right)}^{\sum_{t=1}^{T}y_{t}}\cdot \exp\left\{-\overset{T}{\underset{t=1}{\sum }}{\tilde{\mu }}_{\theta }(t)\right\} \end{array}$$

and the log‐likelihood is given by:

$$l(\theta |y)=\log ℒ(\theta |y)\propto \overset{T}{\underset{t=1}{\sum }}y_{t}\log\left({\tilde{\mu }}_{\theta }(t)\right)-\overset{T}{\underset{t=1}{\sum }}{\tilde{\mu }}_{\theta }(t).$$

Remark that, under the assumption of Poisson distribution and the baseline α=0 (ie, ${\tilde{\mu }}_{\left(\alpha ,\gamma \right)}={\tilde{\lambda }}_{\gamma }(\cdot )$), we can exploit the well‐known Poisson's additive property ‡ to conclude that each cumulative count $Y_{t}^{c}$ is still marginally distributed according to a Poisson, parameterized by the original Richards' curve function $\lambda_{\gamma }(\cdot )$:

$$Y_{t}^{c}|\gamma ∼\text{Pois}\left(\overset{t}{\underset{\tau =1}{\sum }}{\tilde{\lambda }}_{\gamma }(\tau )\right)=\text{Pois}(\lambda_{\gamma }(t)).$$

#### 3.2.2 Negative Binomial distribution

When counts are overdispersed the Poisson distribution is not a suitable choice. We can model the observed daily incidence counts $y=\left\{y_{1},\ldots ,y_{t}\right\}$ as independent realizations from a Negative Binomial with mean ${\tilde{\lambda }}_{\gamma }(t)$ and dispersion parameter $\nu \in {\mathbb{R}}^{+}$:

$$Y_{t}|\theta ∼NB({\tilde{\mu }}_{\theta }(t),\nu ),\;t=1,\ldots ,T.$$

Hence, the likelihood can be written as:

$$\begin{array}{ll} ℒ(\theta ,\nu |d) & =\overset{T}{\underset{t=1}{\prod }}NB(y_{t}|{\tilde{\mu }}_{\theta }(t),\nu )\propto \\ & \propto \overset{T}{\underset{t=1}{\prod }}\left[\frac{\Gamma (\nu +y_{t})}{\Gamma (\nu )}{\left(\frac{\nu }{\nu +{\tilde{\mu }}_{\theta }(t)}\right)}^{\nu }{\left(\frac{{\tilde{\mu }}_{\theta }(t)}{\nu +{\tilde{\mu }}_{\theta }(t)}\right)}^{y_{t}}\right] \end{array}$$

and the log‐likelihood is:

$$\begin{array}{ll} l(\theta ,\nu |y) & =\log ℒ(\theta ,\nu |y)\propto \overset{T}{\underset{t=1}{\sum }}\log\left(\frac{\Gamma (\nu +y_{t})}{\Gamma (\nu )}\right)+\nu \overset{T}{\underset{t=1}{\sum }}\log\left(\frac{\nu }{\nu +{\tilde{\mu }}_{\theta }(t)}\right)\\ & \;+\overset{T}{\underset{t=1}{\sum }}y_{t}\log\left(\frac{{\tilde{\mu }}_{\theta }(t)}{{\tilde{\mu }}_{\theta }(t)+\nu }\right). \end{array}$$

The Negative Binomial does not satisfy the same additive property as the Poisson, hence we cannot draw the same conclusion reached in the Poisson case about the marginal distribution of the cumulative count $Y_{t}^{c}$ when α=0. In general, the cumulative count in the NB case will follow the distribution stemming from the sum of independent Negative Binomial r.v. with common dispersion parameter ν but different means $\tilde{\mu }={\left\{{\tilde{\lambda }}_{\gamma }(t)\right\}}_{t=1}^{T}$.

### 3.3 Response function depending on covariates

The trend of any of the considered indicators may also depend on additional exogenous information, which we may assume to be known *a priori* either because it is immutable (ie, the day of the week), or because policymakers fixed it (daily number of *tested cases*/*swabs* set by the government). For instance: one might want to correct for possible weekly seasonality, which is known to affect the *daily positives* series since many laboratories are closed during the weekend and cannot evaluate swabs. The latter can be used to disentangle the underlying trend of the epidemic from the obvious positive correlation between *tested cases* and *daily positives*. In general, we may want to include the effect of any set of *k* time‐varying covariates $X_{T\times (k+1)}\;={\left[x(t)\right]}_{t=1}^{T}$ in the Richards' framework through the usual linear predictor η(X)=Xβ, where β is a *k* + 1‐dimensional vector of real valued parameters (including intercept). Let us denote the mean function of the considered indicator as ${\tilde{\mu }}_{\theta }(t)=𝔼\left[Y_{t}\right]$, where θ=(α,γ,β). In order to respect the positivity of the mean parameter (which is necessary both in the Poisson and in the Negative Binomial case), we consider the link function g(·)=log(·), so that the effect on the mean is expressed as:

$$\mu_{\beta }\left(X\right)=\exp\left\{\eta (X)\right\}=\exp\left\{X\beta \right\}.$$

Considering a single time point *t*, we would get the following functional form:

$$\mu_{\beta }\left(x(t)\right)=\exp\left\{x(t)\beta \right\}.$$

The mean term of our model shall take into account both the effect of the covariates through $\mu_{\beta }\left(\cdot \right)$ and the temporal behavior induced by the *Richards'* curve $\lambda_{\gamma }(\cdot )$. As a matter of fact, these two components may be combined in different ways. We considered two alternative specifications denoted in the sequel as: *additive* and *multiplicative*.

#### 3.3.1 Additive inclusion of covariates

The inclusion of an additive effect of covariates implies that the effect of every covariate is constant through‐out the pandemic, notwithstanding the current contagion level: for instance, one may think that an increase of daily *tested cases* will always produce the same increase of daily *daily positives*. If that is the case, we may just express the baseline parameter α at each time‐point *t* as the *linked* linear combination of covariates $\mu_{\beta }(x(t))=\exp\left\{x(t)\beta \right\}$, which would produce the following mean function:

$${\tilde{\mu }}_{\theta }(t)=\mu_{\beta }(x(t))+{\tilde{\lambda }}_{\gamma }(t).$$

On the whole vector of observations, this can be expressed as ${\tilde{\mu }}_{\theta }=\mu_{\beta }(X)+{\tilde{\lambda }}_{\gamma }$.

#### 3.3.2 Multiplicative inclusion of covariates

The inclusion of a multiplicative effect of covariates would imply that the more serious the pandemic situation, the more severe the impact of any covariate on the indicators' daily rate.

First, let us recall that in Section 2.1.1 we computed the first differences of the Richards' curve function as:

$${\tilde{\lambda }}_{\gamma }(t)=r\cdot \left[{\left(1+10^{h(p-t)}\right)}^{-s}-{\left(1+10^{h[p-(t-1)]}\right)}^{-s}\right]=r\cdot {\tilde{\lambda }}_{\gamma ,-r}(t).$$

On the log‐scale, it would return the more familiar:

(3) $$\log({\tilde{\lambda }}_{\gamma }(t))=\log(r)+\log\left({\tilde{\lambda }}_{\gamma ,-r}(t)\right).$$

From Equation (3), it comes natural the idea of expressing log(r) at each time‐point *t* as the linear combination of covariates η(x(t)) as in a *Generalized Poisson* model with log link function. Indeed this provides a multiplicative effect of the covariates, where the parameter *r* can be expressed as $\mu_{\beta }(\cdot )$ in the following way:

(4) $$r_{\beta }(x(t))=\mu_{\beta }(x(t))=\exp\left\{x(t)\beta \right\}.$$

Note that the constant *r* is still present and included in Equation (4) through the intercept $\beta_{0}$. Therefore, the mean at time *t* is expressed as:

$${\tilde{\mu }}_{\theta }(t)=\alpha +r_{\beta }(x(t))\cdot {\tilde{\lambda }}_{\gamma ,-r}(t).$$

Considering the whole vector of observations, we would have the following vector of means ${\tilde{\mu }}_{\theta }=\alpha +r_{\beta }(X)\cdot {\tilde{\lambda }}_{\gamma ,-r}$, where $\alpha =\alpha \cdot 1_{T}$.

### 3.4 Model estimation

Parameters can be estimated by maximizing the log‐likelihood l(θ|y), where θ in this case includes all the parameters the likelihood depends on (eg, includes ν in the Negative Binomial case). This optimization problem does not have an analytical solution, and numerical maximization must be used. To improve computation, we derived analytical expressions for the gradient and Hessian of the two possible log‐likelihoods (ie, Poisson or Negative Binomial counts), making Fisher‐scoring iteration very fast. The expressions are reported in the supplementary material. Given the nonsmooth shape of the objective function, we are at risk of being trapped by local maxima of the log‐likelihood, depending on the initial conditions. Therefore, in order to strengthen the optimization procedure, a multistart procedure based on a combination of genetic and gradient descent algorithms has been used.^43^, ^44^

Once an approximate point of maximum $\hat{\theta }$ has been obtained, we could theoretically obtain an estimate of the asymptotic variance‐covariance matrix of the estimated parameters through inverse of the negative log‐likelihood Hessian in $\hat{\theta }$ (which corresponds to the *Observed Fisher Information*):

$${\hat{V}}_{\theta }=-H{\left(l(\hat{\theta }|y)\right)}^{-1},$$

where **H** denotes the Hessian matrix. Nevertheless, we may want to account for the potential misspecification of our model potentially arising from the independence assumption *HI* in Section 3.2. Therefore, we resort to a robust approach for estimating the standard errors and covariance structure associated with the parameter vector θ. In particular, we consider the *Huber Sandwich Estimator* of the variance‐covariance matrix,^45^, ^46^ that can be computed as:

$${\hat{V}}_{\theta }^{R}=\left(-H{\left(l(\hat{\theta }|y)\right)}^{-1}\right)\nabla l(\hat{\theta }|y)\nabla l{\left(\hat{\theta }|y\right)}^{⊤}\left(-H{\left(l(\hat{\theta }|y)\right)}^{-1}\right),$$

where $\nabla l(\hat{\theta }|y)$ represents the gradient of the log‐likelihood in the point of maximum. Interval estimates for the parameters are directly derived through the asymptotic distribution of the *Maximum Likelihood Estimator*, with the corresponding robust covariance matrix $\hat{\theta }∼𝒩\left(\theta ,{\hat{V}}_{\theta }^{R}\right)$. A similar theoretical result for predictions is not as straightforward. Therefore, these are derived through a parametric double bootstrap procedure,^47^, ^48^, ^49^ which accounts for both the uncertainty of parameter estimation and the randomness of the observations. In practice, resampled trajectories ${\left\{Y_{i}\right\}}_{i=1}^{B}$ are obtained by simulating *B* sets of parameters from their asymptotic distribution and computing *B* mean functions trajectories ${\left\{\mu_{\theta_{i}}(t)\right\}}_{i=1}^{B}$. An artificial time series of counts is then simulated for each of the *B* trajectories and 95% confidence intervals are obtained by computing the pointwise 2.5% and 97.5% quantiles. The dispersion parameter ν, being a poorly identifiable nuisance parameter of no impact on the mean curve behavior, has been excluded from the bootstrapping procedure and kept fixed at its estimated value $\hat{\nu }$. Diagnostic check on the model has been performed through the *Pearson residuals* and the *Deviance residuals*. Computation of the former is trivial, where we recall their definition as:

$$\hat{\rho_{t}}=\frac{y_{t}-\hat{y_{t}}}{\hat{\mathrm{Var}}\left[Y_{t}\right]},\;t=1,\ldots ,T.$$

Under the *Poisson* and *Negative Binomial* assumptions we have:

(5) $${\hat{\mathrm{Var}}}_{\text{Poi}}\left[Y_{t}\right]=\mu_{\hat{\theta }}(t),\;{\hat{\mathrm{Var}}}_{\text{NB}}\left[Y_{t}\right]=\mu_{\hat{\theta }}(t)+\frac{\mu_{\hat{\theta }}{\left(t\right)}^{2}}{\hat{\nu }},$$

respectively. The *Deviance Residuals* are instead defined as the individual contributions of each observation to the *Deviance* of the model, that is, the discrepancy between the proposed model and the full model (perfect fit) fits in terms of *log‐likelihood*:

$$\hat{d_{t}}=2\cdot \left[\log\left(f(y_{t}|{\hat{\theta }}_{s}\right)-\log\left(f(y_{t}|\hat{\theta }\right)\right],$$

where *f*(· |·) is the chosen distribution function and ${\hat{\theta }}_{s}$ is the parameter vector of the saturated model. For the *Poisson* and *Negative Binomial* this can be computed as:

$$\begin{array}{ll} & {\hat{d}}_{t}^{\text{Poi}}=\text{sgn}\left(y_{t}-\mu_{\hat{\theta }}(t)\right)\cdot \sqrt{2y_{t}\log\left(\frac{y_{t}}{\mu_{\hat{\theta }}}\right)-\left(y_{t}-\mu_{\hat{\theta }}(t)\right)},\\ & {\hat{d}}_{t}^{\text{NB}}=\text{sgn}\left(y_{t}-\mu_{\hat{\theta }}(t)\right)\cdot \sqrt{2\left[y_{t}\log\left(\frac{y_{t}}{\mu_{\hat{\theta }}(t)}\right)-\left(y_{t}+\nu \right)\cdot \log\left(\frac{y_{t}+\nu }{\mu_{\hat{\theta }}(t)+\nu }\right)\right]}, \end{array}$$

respectively.^50^ If the model correctly describes the variability in the data, then both the *Pearson residuals* and the *Deviance residuals* are expected to be *Normally distributed* and *independent*, with the latter being generally more robust to outliers.

### 3.5 Validation

Fitting performances are further evaluated through numerical metrics such as the pseudo‐R^2^ and coverage of the 95% prediction intervals:

$$\begin{array}{ll} & R^{2}=1-\frac{\text{MSE}}{\sigma_{y}^{2}}=1-\frac{\sum_{t=1}^{T}{\left(y_{t}-{ŷ}_{t}\right)}^{2}}{\sum_{t=1}^{T}{\left(y_{t}-\bar{y}\right)}^{2}},\\ & {\bar{\text{Cov}}}_{95\%}=\frac{1}{T}\cdot \overset{T}{\underset{t=1}{\sum }}{𝕀}_{\left({\hat{y}}_{t}^{l};{\hat{y}}_{t}^{u}\right)}(y_{t}), \end{array}$$

where MSE is the *Mean Squared Error*, $\bar{y}=\frac{1}{T}\sum_{t=1}^{T}y_{t}$ and ${𝕀}_{𝒴}(\cdot )$ denotes the indicator function over the set 𝒴.

### 3.6 Step‐ahead predictions

We test our model's ability to predict the evolution of the epidemic (at least its first wave) from the short to the medium term. Indeed, while the choice of a rigid parametric form for the mean function is penalizing in terms of flexibility and fitting ability, it allows for extrapolation outside the observed domain and is supposed to provide robust forecasts (at least in the short/medium term). Therefore, using the best model for the two indicators (ie, baseline + week‐day additive effect), we calculated the *out‐of‐sample* root mean squared prediction error (RMSPE) for:

- different fitting windows $t=1,\ldots ,\tilde{t}$;
- different forecast horizons, say *K* ∈ {1, 5, 10, 15}.

We recall that, given the fitting window set $1,\ldots ,\tilde{t}$:

$${\text{RMSPE}}_{\tilde{t},K}=\sqrt{\frac{1}{K}\overset{K}{\underset{j=1}{\sum }}{\left(y_{\tilde{t}+j}-{ŷ}_{\tilde{t}+j}\right)}^{2}}.$$

## 4 NOWCASTING THE ITALIAN OUTBREAK OF COVID‐19

For the sake of brevity, here we present results referred to the proposed *Richards' growth model* only for *daily positives* aggregated at the national level. Further results of the model performances for *daily deceased* are included in the supplementary material. We only present results obtained adopting the Negative Binomial distribution because of the substantial overdispersion present at all levels for these indicators (spatially and temporally heterogeneous data collection process, varying containment measures, and so on).

We first show the fitted curve for each indicator, and compare its shape with the observed time series. We also calculate the residuals and check if model assumptions under the proposed framework hold. According to the results drawn from the residual analysis, we modify the empirical setting, keeping the theoretical one fixed, to better capture specific data features.

Later on, we show the performance for two fundamental issues: (i) predicting the epidemic trend in advance and (ii) predicting the *date of the peak* of the epidemic.

### 4.1 Model on *daily positives*

To decide whether or not to include the kink effect, we fitted the model with and without the baseline α and compared the two fits in terms of *log‐likelihood*, *AIC*, *BIC*, and *corrected AIC (AICc)*. The values are presented in Table 1 and provide clear evidence in favor of the model with baseline (ie, with mean $\mu_{\theta }(\cdot )$ as in Equation (2)). Parameters' estimates of the model $\hat{\theta }$ and the respective 95% confidence intervals are shown in Table 2, where the baseline α is estimated to be $\hat{\alpha }=173.17$, with interval (103.2, 290.54), which confirms that the baseline is estimated to be significantly different from 0, and it should be included in the model. As explained in Section 3.1, this parameter represents the long‐term endemic incidence rate that may (possibly indefinitely) follow the end of the main outbreaks. This obviously would hold exactly with constant social interactions, containment measures, control of cases, and so on. Hence, in the considered time horizon, we expect this endemic level to be of ≈173 daily positives per day. When the baseline is included, the parameter *r* does not indicate anymore the final epidemic size, but only the *final outbreak size*. This is the number of positive cases due to the uncontrolled outbreak, additional to what would have been observed in the steady endemic state. This amount is estimated to be ≈222 950, an amount that would have been reached in ≈1288 days at the endemic state level. The parameters *h*, *p*, and *s* do not have an easily quantifiable and absolute interpretation, but are useful for comparison. As explained in Section 3.1, the first indicates how fast the infection spreads, the second how soon it starts descending (lag‐phase) and the last asymmetries between the ascending and descending phase (*s* < 1 the ascending is slower than the descending and viceversa). Finally, ν is an overdispersion parameter and does not present any evident communicable interpretation. The larger it is and the lower the overdispersion, according to the formula in Equation (5).

**TABLE 1:** Log‐likelihood, AIC, BIC, and AICc for the model without baseline and the model with baseline, on daily positives

Index	Model without baseline	Model with baseline
*log‐likelihood*	−1081.4	−982.8
*AIC*	2152.7	1953.6
*BIC*	2162.3	1965
*AICc*	2137.8	1935.7

**TABLE 2:** Parameters' points estimates and 95% confidence intervals for the model with baseline on daily positives

Parameter	Point estimate	95% Interval
α	173.17	(103.2, 290.54)
*r*	222.95 × 10^3^	(220.56 × 10^3^, 225.36 × 10^3^)
*h*	0.0288	(0.0285, 0.0291)
*p*	−31.18	(−32.75, −29.62)
*s*	72.54	(48.29, 96.79)
ν	18.73	(17.77, 19.73)

**FIGURE 5:**
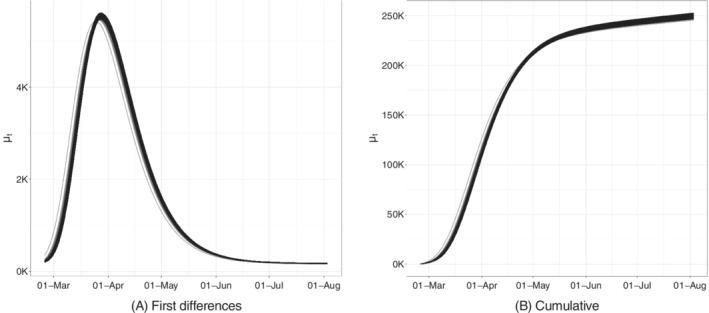
Bootstrapped trajectories corresponding to the Huber Sandwich covariance matrix in the point of maximum for the model with baseline on *daily positives*

We here want to stress the fact that the uncertainty characterizing some of the parameters (like *s*) is not alarming. In particular, variations of *s* at values distant from 1 have very little effect on the curve shape. Furthermore, the parameter vector presents a covariance structure that highlights how different combination of parameters can yield similar curves. Indeed, simulating *M* = 5000 set of parameters from the Normal distribution with variance corresponding to the covariance underlying the Huber Sandwich covariance matrix, we get the set of difference and cumulative curves represented in Figure 5.

We can also directly obtain point predictions ${\left\{{ŷ}_{t}\right\}}_{t=1}^{T}$ as:

(6) $${ŷ}_{t}=\mu_{\hat{\theta }}(t),\;t=1,\ldots ,T,$$

and prediction intervals ${\left\{\left({\hat{y}}_{t}^{l};{\hat{y}}_{t}^{u}\right)\right\}}_{t=1}^{T}$ through the same set of bootstrapped trajectories, whose statistical validity relies on the asymptotic properties introduced in Section 3.4.

**FIGURE 6:**
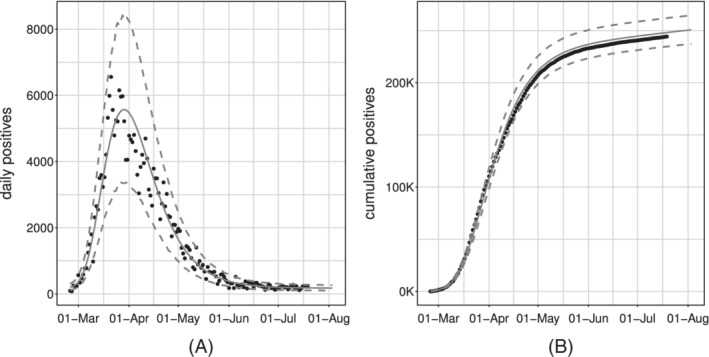
Observed (black dots) and fitted values (gray solid lines) with 95% confidence intervals (gray dashed lines) for the model with baseline on *daily positives*

**FIGURE 7:**
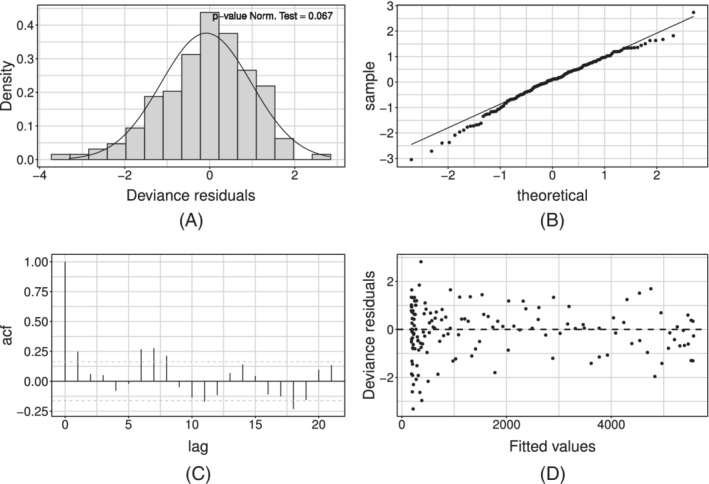
Deviance residuals for the model with baseline on *daily positives*

Figure 6 shows the model fit on the whole available time series of counts: the former on the daily series, the latter on the cumulative one. We can see how the estimated curve does catch the observed general behavior, providing a smooth approximation only marginally influenced by extreme values. Our model produces an R^2^ = 0.941 and coverage ${\bar{\text{Cov}}}_{95\%}=0.945$, meaning that the percentage of observed daily counts falling inside the estimated bounds is perfectly coherent with the specified confidence level. Looking at Figure 6, we notice how daily counts boundaries get smaller as time passes, due to the implicit relationship between mean and variance that characterizes *count* distributions. At the same time, the opposite happens to the bounds on the cumulative counts. The latter is not surprising: indeed, they are built marginally on all the epidemic's possible scenarios. Therefore, they give us a clear sight of what we could have currently observed, keeping into account and aggregating the uncertainty at each stage of the epidemic. We performed a diagnostic check on both the Pearson and the Deviance residuals. The plots in Figure 7 show the Deviance residuals behavior: histogram (A), including the *P*‐value from the Shapiro test; Normal qq‐plot (B); autocorrelation plot (C); plot of the residuals vs fitted values (D). The first two check the (approximated) Normality assumption on the residuals, while the second two control for the correlation of the residuals (among them and with the observed values).

#### 4.1.1 Weekly seasonality

**FIGURE 8:**
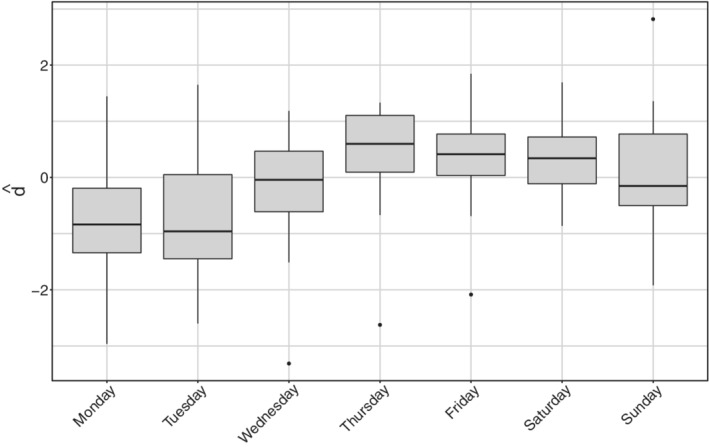
Deviance residuals distribution aggregated by day of the week for *daily positives*

The diagnostic check on both type of residuals showed that the Normality assumption is not rejected, but the correlation plot manifests undesirable patterns (see Figure 7). In particular, the autocorrelation between errors is larger at lag 7 (and multiples of this). We can interpret this outcome as the presence of an intense weekly seasonality (especially during/after the weekend). This suggests people would rather not come forward for testing on the weekend or, alternatively, the system has less capacity at the weekend, meaning it is more challenging to get a test. Undeniably, viruses work 7 days a week. Looking at the low numbers during/after the weekend might give you a false sense of reassurance. This is because it seems cases are down, and therefore elimination of the virus is possible. But, unlike our population, viruses do work on weekends. This may be adjusted by simply adding a weekday effect in our model as a covariate, using the approach in Section 3.3. Such effect may be included either in an additive or a multiplicative fashion. At first, we considered effects for each day of the week, taking *Monday* as a corner point. Preliminary results showed that not all week‐days present a significant deviation from the common mean. On the other hand, the distribution of the Deviance residuals ${\hat{d}}_{t}$ of the standard model aggregated by week‐day (see Figure 8) shows that an evident overestimation pattern (ie, negative deviations) is taking place on Monday and Tuesday.

Therefore, in the sequel, we will present only results obtained with the dichotomous variable that is equal to 1 whenever the week‐day is Monday or Tuesday (0 vice versa). Note that lower tests effort during the weekend shows in the data on Monday and Tuesday, since daily reports involve mostly results received the day before, with swabs therefore dating back 48 hours on the day of publication. This confirms that working with daily data require special care as the cases reporting may suffer from week seasonality. The additive option is chosen over its alternative because of its lower/improved *AIC*, *BIC*, and *AICc* score (see Table 3).

**TABLE 3:** Log‐likelihood, AIC, BIC, and AICc for the models with baseline including additive or multiplicative week‐day effect on daily positives

Index	Additive effect	Multiplicative effect
*log‐likelihood*	−971.74	−974.1
*AIC*	1929.48	1934.3
*AICc*	1942.67	1947.5
*BIC*	1908.60	1913.4

**FIGURE 9:**
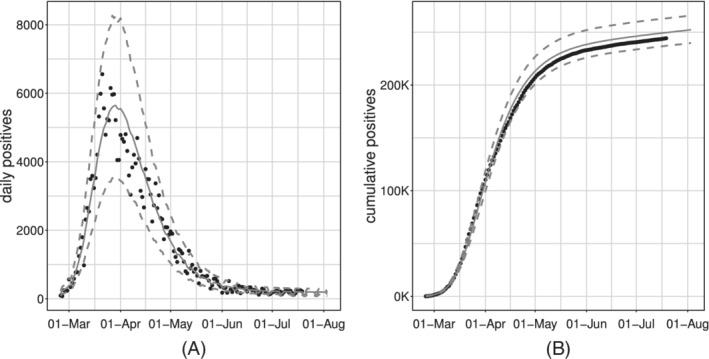
Observed (black dots) and fitted values (gray solid lines) with 95% confidence intervals (gray dashed lines) for the model with baseline and week‐day additive effect, estimated on the *daily positives*

The resulting fit of the model with week seasonality on the observed data are shown in Figure 9, where, on the left, we show the fitted curve and the 95% confidence intervals; on the right, we can observe the fit on the cumulative indicator.

Estimated parameters are shown in Table 4. This model estimates the baseline to be at $e^{{\hat{\beta }}_{0}}=192.48$ on Wednesday to Sunday and at $e^{{\hat{\beta }}_{0}+{\hat{\beta }}_{wd}}=121.51$ on Mondays and Tuesdays. Deriving the corresponding 95% intervals, these two baselines result significantly different from the estimate of the overall baseline $\hat{\alpha }$ in the model without covariates. The estimates of the outbreak size $\hat{r}$ and of the infection rate ĥ of the two models are in agreement, while the point estimates of the asymmetry parameter ŝ are different but both large and mutually included in the corresponding 95% intervals. This is reasonable since we would not expect the outbreak size, rate and symmetry to vary after accounting for week‐day heterogeneity. On the other hand, the new estimate $\hat{p}$ of *p* detects a shorter lag‐phase and hence a slightly faster approach to the descending phase. Finally, the estimate of the dispersion parameter $\hat{\nu }$ is slightly larger than in the model without covariates, denoting less overdispersion with respect to the equivariance hypothesis. This is completely reasonable since the week‐day effect is able to explain some of the previously unaccounted heterogeneity.

**TABLE 4:** Parameters' point estimates and 95% confidence intervals for the additive model on daily positives

Parameter	Point estimate	95% Interval
$\beta_{0}$	5.26	(5.18, 5.34)
$\beta_{wd}$	−0.46	(−0.53, −0.38)
*r*	224.57 × 10^3^	(224.13 × 10^3^, 225.01 × 10^3^)
*h*	0.0289	(0.0287, 0.0291)
*p*	−23.26	(−29.64, −16.88)
*s*	44.42	(−35.67, 124.51)
ν	22.01	(21.35, 22.70)

**FIGURE 10:**
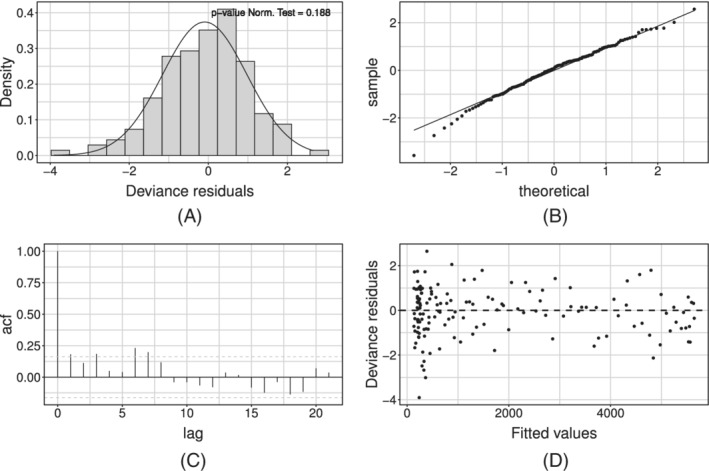
Deviance residuals for the model with baseline and week‐day additive effect estimated on *daily positives*

In terms of model validation, the inclusion of this effect improves sensibly the *R* ^2^ (0.956), while the average coverage ${\bar{\text{Cov}}}_{95\%}$ is constant (0.950). The diagnostic check of the Pearson's and Deviance residuals showed that adherence to Gaussianity improved and the correlation pattern at lag 7 is still present but mitigated (see Figure 10 for the Deviance residuals).

#### 4.1.2 Prediction of future cases and of the peak date

**FIGURE 11:**
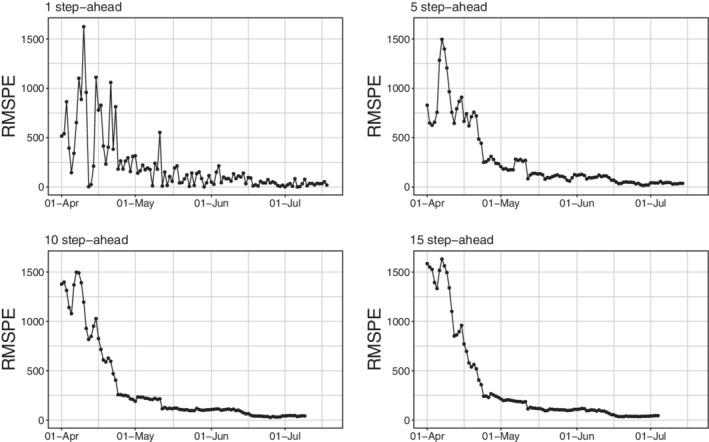
Root mean squared prediction error for *daily positives* at different steps‐ahead

For the latter empirical model, the RMSPEs for each steps‐ahead are presented in Figure 11. Results match the expectations as: (i) the error decreases with the length of the fitting window; (ii) the error trend is more stable on larger testing windows (10‐15 steps ahead vs 1‐5 steps ahead); (iii) larger errors are made around the day of the peak. It can be seen nevertheless that predictions are always reasonable at these time horizons. This is a good point for our theoretical framework, as it can be used as a guidance tool to plan nonpharmaceutical‐interventions due to its capability to predict future scenarios with reasonable accuracy. Of course, with more detailed data and including confounding factors, the accuracy may be further improved. Unfortunately, the aggregated available data do not contain important information which may strongly improve the prediction, for example, stratifications of cases by age, gender, comorbidities, and so on.

**FIGURE 12:**
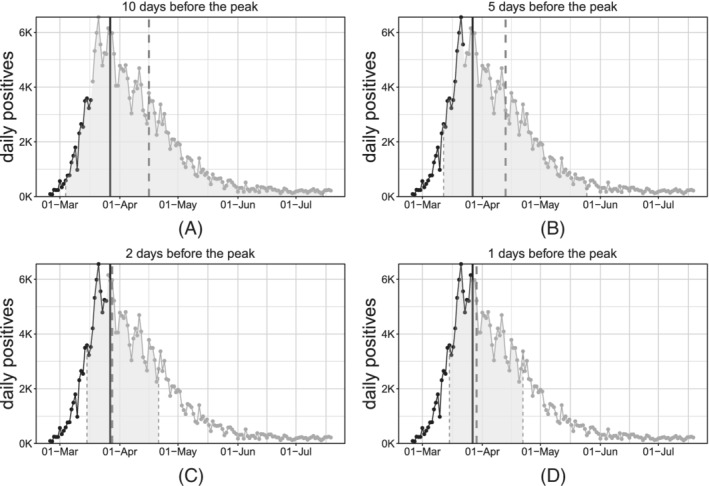
Estimation of the date of the peak for *daily positives* at different steps‐before

Finally, we evaluate the model's ability to predict the date of the peak. The approximate dates and heights of the peak have important epidemiological implications. This becomes possible under the assumption that sensible modifications of the adopted epidemiological strategies do not emerge. However, if exogenous events, for example, efficient treatments or vaccines, arise at a certain point in time, our framework allows to include it to predict the peak, in a similar manner as we did for the week seasonality effect. To do so, we estimate the model without covariates, using all available data until *K* ∈ {15, 10, 5, 3, 2, 1} days before the observed peak. For the sake of conciseness, we only report results for *K* ∈ {10, 5, 2, 1} as shown in Figure 12.

When *s* = 1, the peak $\hat{t}$ is directly expressed by the parameter *p*. When *s* ≠ 1, after some algebra it can be seen that the peak can still be computed analytically as:

$$\hat{t_{\hat{\gamma }}}=\hat{p}+\frac{\log_{10}(ŝ)}{ĥ}.$$

Confidence intervals are obtained through the same bootstrap procedure introduced in Section 3.4. The dashed gray vertical lines represent the bounds of the confidence interval and the predicted date of the peak (confidence area is shaded with the same gray). The solid vertical black line represents the *“true”* date of the peak (ie, obtained via smoothing of the observed counts through nonparametric polynomial approximations). The observed time‐series is represented through point and lines, where the black section is referred to the training window while the gray section is referred to the testing (out‐of‐sample) window.

As expected, as we approach the real date of the peak, we predict it more accurately. Point predictions are very accurate since 5 days before the actual peak. At the same time, interval bounds get tighter and tighter as the fitting interval approached the day of the peak and, in general, the day of the peak is always included in such bounds (see Table 5 for exact numerical evaluation).

**TABLE 5:** Delay (days) in point estimation of the peak

	10		5		2		1	
Days before	Delay	Width	Delay	Width	Delay	Width	Delay	Width
*daily deceased*	−1	37	–3	25	–4	22	–3	21
*daily positives*	20	106	17	69	1	37	2	37

All the aforementioned results have been calculated also for the national aggregated *daily deceased*. Exposition and discussion of these results, which are in fact very similar to the *daily positives*ones, are included in the supplementary material. Here, we just want to highlight how the peak is accurately predicted with a shorter delay and generally smaller uncertainty for the *daily deceased* than for the *daily positives* (see Table 5). This is probably related to the more regular behavior of the series, due to a likely more homogeneous collection process of the records.

Finally, we here want to stress the point that we are introducing a framework with the highly desirable goal to formulate a model which would predict an evolution curve. To be more precise, a great variety of epidemiological models have been proposed in the literature, but most standard versions of SIR‐like models typically yield an increase before the peak that is quite similar to the decrease after the peak. The proposed framework, based on more complex evolution dynamics, is robust enough to be fitted successfully on the (poor quality) available data while explaining and forecasting different increasing and decreasing behavior before and after the peak. We emphasize that such increase‐decrease quantitative behaviors appear to satisfactorily conform to reality.

## 5 DISCUSSION AND FURTHER WORK

We presented an approach to modeling and prediction of epidemic indicators that has proven useful during the first outbreak of COVID‐19 in Italy. The model has been validated on publicly available data, and has proved flexible enough to adapt to different indicators.

It is important to underline up front that the available data are clearly biased. Incidence depends on testing and tracing efforts, whose indications have varied wildly over time and space. Comparability of indicators over time and space might in part be achieved by including the daily number of swabs as a predictor, which anyway would make predictions cumbersome. Different definitions of COVID‐19 related death make it also very hard to compare mortality across countries. This problem does not apply to our data, that refer only to Italy. However, while this definition has been constant over time in Italy, it shall be remarked that also deaths might be underestimated, with the degree of undercount positively associated with incidence. Correcting for this bias is not trivial, and would require corrections based on individual‐level data and/or reliable statistics about excess mortality.

Summarizing the results, we would like to emphasize that the proposed Richards' curve model describes properly the growth in the number of COVID‐19 daily positives and daily deceased, despite its simplicity. Indeed, it is able to reflect properly the trend of the daily incidence indicators; and also allows for the straightforward inclusion of exogenous information. Basic covariates such as the week‐day effect proved to sensibly enhance model fitting and prediction accuracy. While we have illustrated results at the national level, the model can be used also at the regional/local level (perhaps including specific local effects). The resulting fits are included in the supplementary material. The maximum likelihood approach so far considered is rather stable, as long as reasonable starting values are found to initialize the algorithm.

A limitation of our approach is that logistic growth curves are constrained so that only one wave at a time can be successfully modeled. This implies that initial (and possibly final) dates shall be set by the user to identify a wave. This is rather simple empirically (eg, the initial date can be the last day with zero incidence, and the final date can be the first day with incidence above (or under) a prespecified threshold). On the other hand, multiple waves could be modeled by modification of our nonlinear model as a weighted average of multiple Richards' curves (one for each wave), in which weights of the noncurrent wave are forced to decay to zero with the distance from the wave‐specific peak. We leave this as grounds for further work.

A Bayesian approach will also be experimented in order to overcome possible issues with the asymptotic properties of the maximum likelihood estimator. Notably, implementation of the *no‐U‐turn sampler* algorithm for the estimation of nonlinear models might be a valid working solution. In addition, a Bayesian approach may also be used to include spatial dependence into the modeling framework and also to relax the first‐order Markov assumption for taking into account more complex temporal dependence. In particular, the latter may be key in order to adapt the introduced Richards' curve model for the nowcasting of prevalence indicators, for example, *current positives* and *current intensive care units occupancy*. Indeed, any modeling effort shall account for the strong temporal dependence between subsequent counts stemming from the fact that daily counts at time *t* potentially include units which are in stock since times τ<t. Furthermore, as specified in Section 2.1.2, prevalence indicators are nonmonotonic and their value is the result of the combination of the incidence components building up each of those. These two last issues may be addressed by adapting the Richards' response function to accommodate nonmonotonicity and/or by hierarchically specifying a model for the prevalence indicators through the combination of models for their incidence components. A successful attempt in accurately nowcasting the *ICU occupancy* is given in Reference 51.

## SOFTWARE

Software in the form of R code, together with a sample input dataset and complete documentation is available on request from the corresponding author.

## Supporting information

Appendix S1

## Data Availability

The data that support the findings of this study are openly available in “Protezione Civile Italiana GitHub Repository” at https://github.com/pcm‐dpc/COVID‐19.

## Footnotes

* see https://www.epiprev.it/materiali/2020/EP2‐3/112_edit1.pdf for further details.

† Conversely, we may assume *h*, *s* < 0

‡ the sum of independent Poissons is still a Poisson with parameter the sum of the parameters
