# Supplementary material for: Nowcasting COVID‐19 incidence indicators during the Italian first outbreak
Source: Stat Med. 2021 May 6;40(16):3843–64. doi: 10.1002/sim.9004 (PMC8242495; doi:10.1002/sim.9004)
Supplement: Supplementary file 1 — Appendix S1 [file SIM-40-3843-s001.pdf]

ORIGINAL ARTICLE

Nowcasting COVID-19 incidence indicators during the Italian first outbreak

Pierfrancesco Alaimo Di Loro[1] | Fabio Divino[2] | Alessio Farcomeni[3] | Giovanna Jona Lasinio[1] | Gianfranco Lovison[4,5] | Antonello Maruotti[6,7] | Marco Mingione[1,8]

[1, *]University of Rome "La Sapienza", Dpt. of Statistical Sciences

[2]University of Molise, Dpt. of Bio-Sciences

[3]University of Rome "Tor Vergata", Dpt. of Economics and Finance

[4]University of Palermo, Dpt. of Economics, Management and Statistics

[5]Swiss TPH Basel, Dpt. of Epidemiology and Public Health

[6]Libera Università Maria Ss Assunta, Dpt. GEPLI

[7]University of Bergen, Dpt. of Mathematics

[8]Institute of Applied Computing "M. Picone", IAC - CNR

Correspondence
Dpt. of Statistical Sciences, University of Rome "La Sapienza", Rome, 00185, Italy
Email:
pierfrancesco.alaimodiloro@uniroma1.it

Present address
[*]Dpt. of Statistical Sciences, University of Rome "La Sapienza", Rome, Lazio, 00185, Italy

A novel parametric regression model is proposed to fit incidence data typically collected during epidemics. The proposal is motivated by real time monitoring and short-term forecasting of the main epidemiological indicators within the first outbreak of COVID-19 in Italy. Accurate short-term predictions, including the potential effect of exogenous or external variables are provided; this ensures to accurately predict important characteristics of the epidemic (e.g., peak time and height), allowing for a better allocation of health resources over time. Parameters estimation is carried out in a maximum likelihood framework. All computational details required to reproduce the approach and replicate the results are provided.

Supplementary material

Gradients

In order to make the optimization procedure robust, gradients and Hessians used for the estimation (optimization routine on the log-likelihood) have been computed analytically. This section provides insights about their derivation for the log-likelihoods at hand. For the sake of clarity, in the sequel, we will invert the previous notation and denote the functions of interest as functions of the parameters, given the observed time points: e.g. $\tilde{\mu}_{\boldsymbol{\theta}}(t)$ becomes $\tilde{\mu}_t(\boldsymbol{\theta})$. We first provide the computations for the gradient of the log-likelihood for both Poisson and Negative Binomial distributions by considering their mean function $\tilde{\mu}_t(\boldsymbol{\theta})$ as a whole. Afterwards, we show the gradients and introduce the Hessians specific to $\tilde{\lambda}_t(\gamma)$, as it is the most cumbersome component of the mean to derive with respect to its parameters.

| Poisson Gradient

Let q denote any of the elements of $\boldsymbol{\theta}$, vector of parameters characterizing the mean function $\tilde{\mu}_t(\boldsymbol{\theta})$. The generic derivative with respect to the component q of $\boldsymbol{\theta}$ for the Poisson log-likelihood Poi$(\tilde{\mu}_t(\boldsymbol{\theta}))$ is:

$$\begin{aligned}\frac{\partial}{\partial q} l_{Poi}(\gamma|\mathbf{y}) &= -\sum_{t=1}^{T} \frac{\partial}{\partial q}\tilde{\mu}_t(\boldsymbol{\theta}) + \sum_{t=1}^{T} y_t \frac{\partial}{\partial q} \log(\tilde{\mu}_t(\boldsymbol{\theta})) = \\ &= -\sum_{t=1}^{T} \frac{\partial}{\partial q}\tilde{\mu}_t(\boldsymbol{\theta}) + \sum_{t=1}^{T} y_t \frac{1}{\tilde{\mu}_t(\boldsymbol{\theta})} \frac{\partial}{\partial q}\tilde{\mu}_t(\boldsymbol{\theta}).\end{aligned} \tag{1}$$

| Negative Binomial Gradient

The Negative Binomial NB $(\nu, \mu_t(\boldsymbol{\theta}))$ presents the additional parameter ν, which does not affect the mean function but controls for the dispersion. In the following, we provide the first derivative with respect to ν and with respect to the generic element q of $\boldsymbol{\theta}$, respectively.

The first derivative with respect to ν of the log-likelihood is:

$$\begin{aligned}\frac{\partial}{\partial \nu} l_{NB}(\nu, \boldsymbol{\theta}|\mathbf{y}) &= T(\log(\nu) - \psi(\nu)) + \\ &+ \sum_{t=1}^{T} \left(\psi(\nu + y_t) - log\ (\mu_t(\boldsymbol{\theta}) + \nu) + \frac{\mu_t(\boldsymbol{\theta}) - y_t}{\mu_t(\boldsymbol{\theta}) + \nu} \right)\end{aligned} \tag{2}$$

where $\psi(\cdot)$ denotes the *digamma* function. The generic derivative with respect to q of the log-likelihood is:

$$\begin{aligned}\frac{\partial}{\partial q} l_{NB}(\nu, \gamma|\mathbf{y}) &= -\sum_{t=1}^{T} \frac{y_t + \nu}{\tilde{\mu}_t(\boldsymbol{\theta}) + \nu} \frac{\partial}{\partial q}\tilde{\mu}_t(\boldsymbol{\theta}) + \\ &+ \sum_{t=1}^{T} \frac{y_t}{\tilde{\mu}_t(\boldsymbol{\theta})} \frac{\partial}{\partial q}\tilde{\mu}_t(\boldsymbol{\theta}).\end{aligned} \tag{3}$$

| Richards' Gradient

Derivation of the gradient $\tilde{\mu}_t(\boldsymbol{\theta})$ can be obtained by deriving separately (but appropriately) each of the pieces composing it. Computations are straightforward for all components, but for the Richards' first differences parameters, which can in turn be divided as:

$$\frac{\partial}{\partial \gamma_i}\tilde{\lambda}_t(\gamma) = \frac{\partial}{\partial \gamma_i}\lambda_t(\gamma) - \frac{\partial}{\partial \gamma_i}\lambda_{t-1}(\gamma)$$

The Richards' function gradient is composed of the following four terms:

$$\nabla \lambda_t(\gamma) = \left[\frac{\partial}{\partial r}\lambda_t(\gamma),\ \frac{\partial}{\partial h}\lambda_t(\gamma),\ \frac{\partial}{\partial p}\lambda_t(\gamma),\ \frac{\partial}{\partial s}\lambda_t(\gamma)\right]^\top$$

which can be computed as follows.

$$\frac{\partial}{\partial r}\lambda_t(r) = \frac{\partial}{\partial r}\left(b + \frac{r}{(1+10^{h(p-t)})^s}\right) = \frac{1}{(1+10^{h(p-t)})^s},$$

$$\begin{aligned}\frac{\partial}{\partial h}\lambda_t(h) &= \frac{\partial}{\partial h}\left(b + \frac{r}{(1+10^{h(p-t)})^s}\right) = \\ &= -r \cdot s \cdot (1+10^{h(p-t)})^{-s-1} 10^{h(p-t)} (p-t) \log(10),\end{aligned}$$

$$\begin{aligned}\frac{\partial}{\partial p}\lambda_t(p) &= \frac{\partial}{\partial p}\left(b + \frac{r}{(1+10^{h(p-t)})^s}\right) = \\ &= -r \cdot s \cdot (1+10^{h(p-t)})^{-s-1} 10^{h(p-t)} h \log(10),\end{aligned}$$

$$\begin{aligned}\frac{\partial}{\partial s}\lambda_t(s) &= \frac{\partial}{\partial s}\left(b + \frac{r}{(1+10^{h(p-t)})^s}\right) = \\ &= -r \cdot \left(1+10^{h(p-t)}\right)^{-s} \log\left(1+10^{h(p-t)}\right)\end{aligned}$$

| Log-scale

In the R implementation, the log-likelihood has been parametrized on the log-scale for all the parameters defined on $\mathbb{R}^+$ in order to ease the optimization process under the positivity constraint. This means that given $q \in \{b, r, p, s\}$, the log-likelihood uses $\log(q) = v$, where $q = e^v$. This implies that, when we differentiate, we have to take into account the Jacobian as a result of the transformation:

$$\frac{\partial}{\partial v}\lambda_t(\gamma) = \frac{\partial}{\partial e^v}\lambda_t(\gamma)\frac{\partial e^v}{\partial v} = \frac{\partial}{\partial e^v}\lambda_t(\gamma) \cdot e^v = \frac{\partial}{\partial q}\lambda_t(\gamma) \cdot q. \tag{4}$$

Therefore, each derivative must be multiplied by $e^v = q$.

Hessians

Hessians used for the estimation procedure of the model (optimization routine on the log-likelihood) have been computed analytically. In the sequel, we first provide the Hessian for the log-likelihod of Poisson and Negative Binomial by considering $\lambda_t(\gamma)$ as a whole.

| Poisson Hessian

Let q and f denote any pair of the parameters characterizing the mean function $\tilde{\mu}_t(\boldsymbol{\theta})$.

The mixed second derivative with respect to the components q and f of $\boldsymbol{\theta}$ for the Poisson log-likelihood is:

$$\frac{\partial^2}{\partial xf} l_{Poi}(\boldsymbol{\theta}|\mathbf{y}) = \sum_{t=1}^{T} \frac{y_t - \tilde{\mu}_t(\boldsymbol{\theta})}{\tilde{\mu}_t(\boldsymbol{\theta})} \frac{\partial^2}{\partial qf}\tilde{\mu}_t(\boldsymbol{\theta}) - \sum_{t=1}^{T} \frac{y_t}{\tilde{\mu}_t(\boldsymbol{\theta})^2} \frac{\partial}{\partial q}\tilde{\mu}_t(\boldsymbol{\theta}) \frac{\partial}{\partial f}\tilde{\mu}_t(\boldsymbol{\theta}).$$

The second derivative with respect to the components q for the Poisson log-likelihood is:

$$\frac{\partial^2}{\partial q^2} l_{Poi}(\boldsymbol{\theta}|\mathbf{y}) = \sum_{t=1}^{T} \frac{y_t - \tilde{\mu}_t(\boldsymbol{\theta})}{\tilde{\mu}_t(\boldsymbol{\theta})} \frac{\partial^2}{\partial q^2}\tilde{\mu}_t(\boldsymbol{\theta}) - \sum_{t=1}^{T} \frac{y_t}{\tilde{\mu}_t(\boldsymbol{\theta})^2} \left(\frac{\partial}{\partial q}\tilde{\mu}_t(\boldsymbol{\theta})\right)^2.$$

| Negative Binomial Hessian

Let q and f denote any pair of the parameters characterizing the mean function $\mu_t(\boldsymbol{\theta})$.

The mixed second derivative with respect to q and f of the Negative Binomial log-likelihood is:

$$\begin{aligned}\frac{\partial^2}{\partial xf} l_{NB}(\boldsymbol{\theta}|\mathbf{y}) = &\sum_{t=1}^{T} \left(\frac{y_t + \nu}{(\mu_t(\boldsymbol{\theta}) + \nu)^2} - \frac{y_t}{\mu_t(\boldsymbol{\theta})^2}\right) \frac{\partial}{\partial q}\tilde{\mu}_t(\boldsymbol{\theta}) \frac{\partial}{\partial f}\tilde{\mu}_t(\boldsymbol{\theta}) \quad + \\ &+ \sum_{t=1}^{T} \left(\frac{y_t}{\tilde{\mu}_t(\boldsymbol{\theta})} - \frac{y_t + \nu}{\tilde{\mu}_t(\boldsymbol{\theta}) + \nu}\right) \frac{\partial^2}{\partial qf}\tilde{\mu}_t(\boldsymbol{\theta}).\end{aligned}$$

The second derivative with respect to q of the Negative Binomial log-likelihood is:

$$\begin{aligned}\frac{\partial^2}{\partial q^2} l_{NB}(\boldsymbol{\theta}|\mathbf{y}) = &\sum_{t=1}^{T} \left(\frac{y_t + \nu}{(\tilde{\mu}_t(\boldsymbol{\theta}) + \nu)^2} - \frac{y_t}{\tilde{\mu}_t(\boldsymbol{\theta})^2}\right) \left(\frac{\partial}{\partial q}\tilde{\mu}_t(\boldsymbol{\theta})\right)^2 \quad + \\ &+ \sum_{t=1}^{T} \left(\frac{y_t}{\tilde{\mu}_t(\boldsymbol{\theta})} - \frac{y_t + \nu}{\tilde{\mu}_t(\boldsymbol{\theta}) + \nu}\right) \frac{\partial^2}{\partial q^2}\tilde{\mu}_t(\boldsymbol{\theta}).\end{aligned}$$

In the Negative Binomial case, we must recall the presence of the additional parameter ν. The second derivative with respect to ν of the Negative Binomial log-likelihood is:

$$\begin{aligned}\frac{\partial^2}{\partial \nu^2} l_{NB}(\boldsymbol{\theta}|\mathbf{y}) = &T\left(\frac{1}{\nu} - \psi'(\nu)\right) + \\ &+ \sum_{t=1}^{T} \left(\psi'(\nu + y_t) - \frac{1}{\tilde{\mu}_t(\boldsymbol{\theta}) + \nu} - \frac{\tilde{\mu}_t(\boldsymbol{\theta}) - y_t}{(\tilde{\mu}_t(\boldsymbol{\theta}) + \nu)^2}\right)\end{aligned}$$

where $\psi(\cdot)$ and $\psi'(\cdot)$ denote the *digamma* and the *trigamma* function, respectively.

The mixed derivative with respect to ν and the generic element q of γ is:

$$\frac{\partial^2}{\partial \nu q} l_{NB}(\gamma|\mathbf{y}) = \sum_{t=1}^{T} \frac{y_t - \tilde{\mu}_t(\boldsymbol{\theta})}{(\tilde{\mu}_t(\boldsymbol{\theta}) + \nu)^2} \frac{\partial}{\partial q} \tilde{\mu}_t(\boldsymbol{\theta})$$

| Richards' Hessian

As for the gradient, the same holds for the Hessian of the first differences of the Richards function, which would be only one interesting computation to show. Also here:

$$\frac{\partial^2}{\partial \gamma_i \gamma_j} \tilde{\lambda}_t(\gamma) = \frac{\partial^2}{\partial \gamma_i \gamma_j} \lambda_t(\gamma) - \frac{\partial^2}{\partial \gamma_i \gamma_j} \lambda_{t-1}(\gamma)$$

In particular, the resulting Hessian is a 4×4 matrix such that:

$$[\mathbf{H}(\lambda_t(\gamma))]_{ij} = \frac{\partial^2}{\partial \gamma_i \gamma_j} \lambda_t(\gamma), \quad i, j \in \{1, \ldots, 4\}.$$

Computations are straightforward for most of the terms, but the final result counts 10 terms (the Hessian matrix is symmetric) and some of those terms are cumbersome to report. Therefore, we won't include these here. The reader, if interested, is invited to contact the authors for further details on their detailed computation.

| Log-scale

In the R implementation, the log-likelihood has been parametrized on the log-scale for all the parameters defined on $\mathbb{R}^+$ in order to ease the optimization process under the positivity constraint. This means that given two generic elements, say q and f, of the parameters' vector γ, the log-likelihood uses $\log(q) = v$ and $\log(f) = u$, where $q = e^v$ and $f = e^u$. The Jacobian inclusion has two implications on the Hessian. When computing the mixed derivative, we need to account for the transformation of both terms (if both are on the log scale):

$$\begin{aligned}
\frac{\partial^2}{\partial v \partial u} \lambda_t(\gamma) &= \frac{\partial}{\partial v} \left(\frac{\partial}{\partial u} \lambda_t(\gamma) \right) = \frac{\partial}{\partial v} \left(\frac{\partial}{\partial e^u} \lambda_t(\gamma) \frac{\partial e^u}{\partial u} \right) = \\
&= \frac{\partial}{\partial v} \left(\frac{\partial}{\partial e^u} \lambda_t(\gamma) \cdot e^u \right) = \frac{\partial}{\partial e^v} \left(\frac{\partial}{\partial e^u} \lambda_t(\gamma) \cdot e^u \right) \frac{\partial e^v}{\partial v} = \\
&= \frac{\partial}{\partial e^v} \left(\frac{\partial}{\partial e^u} \lambda_t(\gamma) \cdot e^u \right) \cdot e^v = \frac{\partial}{\partial q} \frac{\partial}{\partial s} \lambda_t(\gamma) \cdot q \cdot f.
\end{aligned} \tag{5}$$

Therefore, each mixed derivative $\frac{\partial^2}{\partial xs}\lambda_t(\gamma)$ must be multiplied by both $e^v = q$ and $e^u = f$. When computing the second derivative for v, we need to recall that the first derivative contains the Jacobian, so:

$$\begin{aligned}
\frac{\partial^2}{\partial v}\lambda_t(\gamma) &= \frac{\partial}{\partial v}\left(\frac{\partial}{\partial v}\lambda_t(\gamma)\right) = \frac{\partial}{\partial e^v}\left(\frac{\partial}{\partial e^v}\lambda_t(\gamma)\cdot e^v\right)\cdot e^v = \\
&= \left(\frac{\partial}{\partial e^v}\frac{\partial}{\partial e^v}\lambda_t(\gamma)\cdot e^v + \frac{\partial}{\partial e^v}\lambda_t(\gamma)\cdot\frac{\partial}{\partial e^v}e^v\right)\cdot e^v = \\
&= \left(\frac{\partial}{\partial q}\frac{\partial}{\partial q}\lambda_t(\gamma)\cdot q + \frac{\partial}{\partial q}\lambda_t(\gamma)\cdot\frac{\partial}{\partial q}q\right)\cdot q = \\
&= \frac{\partial^2}{\partial q^2}\lambda_t(\gamma)\cdot q^2 + \frac{\partial}{\partial q}\lambda_t(\gamma)\cdot q.
\end{aligned} \tag{6}$$

Model on *daily deceased*

The same analysis carried out for the *daily positives* has been conducted on the *daily deceased*. Comparisons in terms of goodness of fit measures are reported for both models in Table 1. The best model in terms of all the goodness of fit scores (AIC, AICc and BIC) is the model with baseline. The resulting estimated parameters $\hat{\boldsymbol{\theta}}$ and the respective intervals are shown in Table 2, where the baseline α is estimated to be $\hat{\alpha} = 3.3$ (sensibly larger than 0). Hence, in the considered time horizon, we expect the endemic fatality rate to be of ≈ 3 deaths per day. The final outbreak size r is estimated to be $\approx 35,810$, an amount that would have been reached in $\approx 10,851$ days at the endemic fatality rate. As for the daily positives, the parameters h, p, s and v do not have an easily quantifiable and absolute interpretation, but are useful for comparisons.

TABLE 1 *Log-likelihood, AIC, BIC and AICc for the model without baseline and the model with baseline on daily deceased*

**Index**	Model without baseline	Model with baseline
log-likelihood	−735.8	−732.5
AIC	1461.6	1452.9
AICc	1471.2	1464.4
BIC	1446.7	1435.1

TABLE 2 *Parameters' points estimates and 95% confidence intervals for the model with baseline on daily deceased*

**Parameter**	Point estimate	95% Interval
α	3.3	$(1.97, 5.54)$
r	35.73×10^3	$(35.34 \times 10^3, 36.11 \times 10^3)$
h	0.0247	$(0.0244, 0.0249)$
p	−50.50	$(-52.07, -48.93)$
s	171.58	$(130.33, 201.83)$
v	12.36	$(11.73, 13.02)$

We can then obtain point predictions $\{\hat{y}_t\}_{t=1}^T$ and prediction intervals $\left\{(\widehat{y}_t^l; \widehat{y}_t^u)\right\}_{t=1}^T$ through the parametric bootstrap procedure described in the Main Text. Fig. 1 shows the fit on the whole available time series of counts: the former on the daily series, the latter on the cumulative one. Also in the case of the deceased the estimated curve does catch the observed general behavior. The same metrics are used to evaluate the fitting performances, which correspond to an $R^2 = 0.90$ and a coverage $\overline{\text{Cov}}_{95\%} = 0.95$. Also here, we performed a diagnostic check on both the Pearson and the Deviance residuals. The plots in Fig. 2 show the Deviance residuals behavior: histogram (a), including the p-value from the Shapiro test; Normal qq-plot (b); auto-correlation plot (c); plot of the residuals vs. fitted values (d).

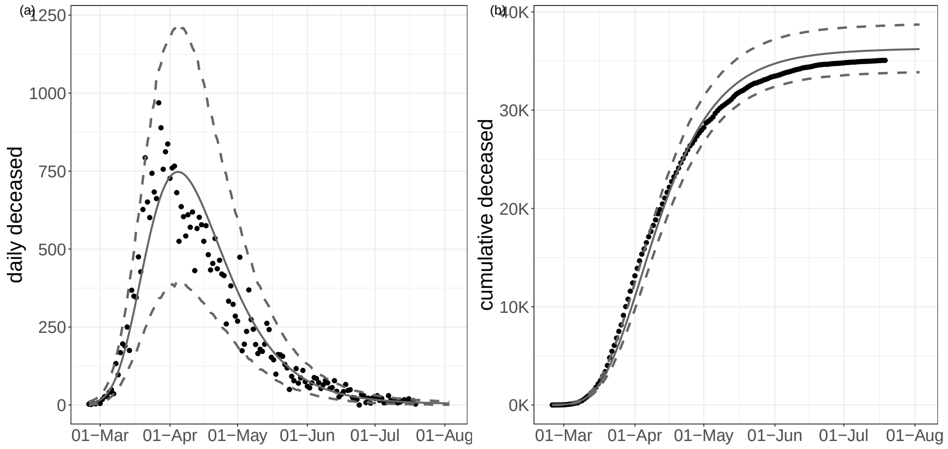

FIGURE 1 Observed (black dots) and fitted values (grey solid lines) with 95% confidence intervals (grey dashed lines) for model with baseline on *daily deceased*.

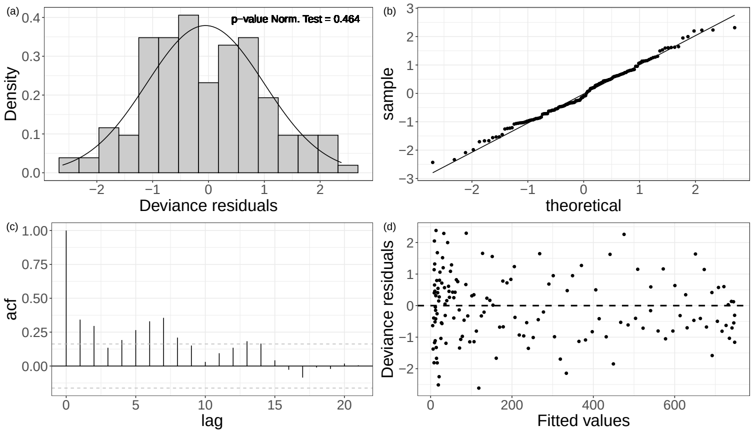

FIGURE 2 Deviance residuals for the model with baseline on *daily deceased*.

| Weekly seasonality

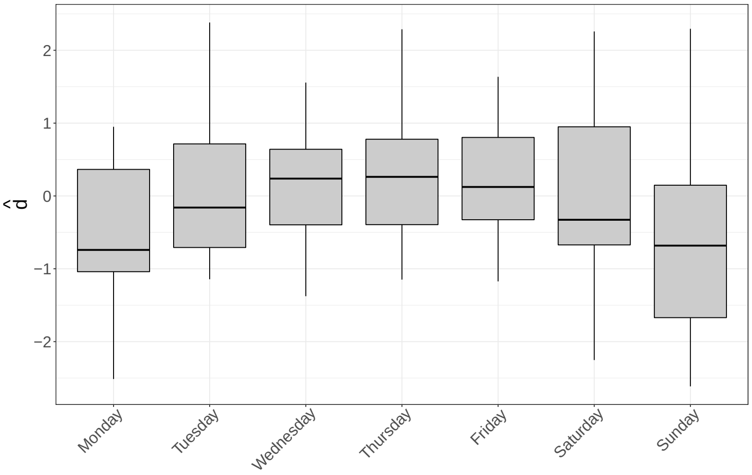

FIGURE 3 Deviance residuals distribution aggregated by day of the week for *daily deceased*.

As in the case of *daily positives*, the diagnostic check on the residuals for the *daily deceased* highlights a slight week seasonality pattern for the auto-correlations. In addition, also the residual Normality hypothesis is rejected. Potentially, the inclusion of a week-day effect may solve both problems. In order to decide what set of week-days to group together, we visualize the residuals' distribution aggregated by week (see Fig. 3). The pattern is not as evident as in the case of *daily positives*, but we can still detect some undesirable overestimation on Mondays and Sundays.

TABLE 3 *Log-likelihood, AIC, BIC and AICc for the models with baseline including additive or multiplicative week-day effect on daily deceased*

**Index**	Additive effect	Multiplicative effect
log-likelihood	−725.77	−725.30
AIC	1437.78	1436.61
AICc	1450.97	1449.81
BIC	1416.90	1415.73

Therefore, on the line of the previous application, we decide to include a dichotomous week-day fixed effect on the pair Sunday-Monday. As before, this effect may be included either in an additive or a multiplicative fashion and, again, we may pick the version that achieves the best AIC, AICc and BIC scores. However, as shown in Table 3, differences in these scores are almost negligible and choice based on such a small improvement would not be robust. Therefore, we checked the Pearson residuals for both alternatives and we selected the additive model because of the improved residuals behavior (Normality is accepted, autocorrelation at lag 7 is reduced). The resulting fit is shown in Fig. 4 where: on the left, we can observe the fitted curve and the 95% confidence intervals; on the right, we can

TABLE 4 *Intercept β_0 and week-day effect β_{wd} point estimates and 95% confidence intervals for the additive model with baseline on daily deceased*

**Parameter**	Point estimate	95% Interval
β_0	1.85	(1.73, 1.98)
β_{wd}	−510.36	(−573.84, −446.88)
r	35.81×10^3	$(33.58 \times 10^3, 38.19 \times 10^3)$
h	0.0251	(0.0246, 0.0255)
p	−58.88	(−63.52, −54.22)
s	297.24	(199.12, 390.36)
ν	13.58	(10.01, 18.41)

observe the cumulative fit. Estimated parameters are shown in Table 4, where the Sunday-Monday effect is estimated to have a strong reducing effect on the daily baseline rate of ≈ -510 on the log-scale, i.e. $\exp\{-510\} \approx 0$, which shrinks to 0 the *baseline* on Mondays and Sundays. The estimates of the outbreak size $\hat{r}$ and of the infection rate $\hat{h}$ of the two models are in agreement, while the point estimates of the asymmetry parameter $\hat{s}$ are different but both large and mutually included in the corresponding 95% intervals. This is reasonable since we would not expect the outbreak size, rate and symmetry to vary wildly after accounting for week-day heterogeneity. On the other hand, the new estimate $\hat{p}$ of p detects a longer lag-phase and hence a slightly slower approach to the descending phase. Finally, the estimate of the dispersion parameter $\hat{\nu}$ is slightly larger than in the model without covariates, denoting less over-dispersion with respect to the equi-variance hypothesis. This is completely reasonable since the week-day effect is able to explain some of the previously unaccounted heterogeneity. The inclusion of the Sunday-Monday effect allows for an increase of the R^2 to 0.91, whilst keeping the coverage $\overline{\text{Cov}}_{95\%}$ steady at 0.95. The diagnostic check shown in Fig. 5 shows how Residual Normality is now accepted and the previously evident correlation pattern is slightly reduced.

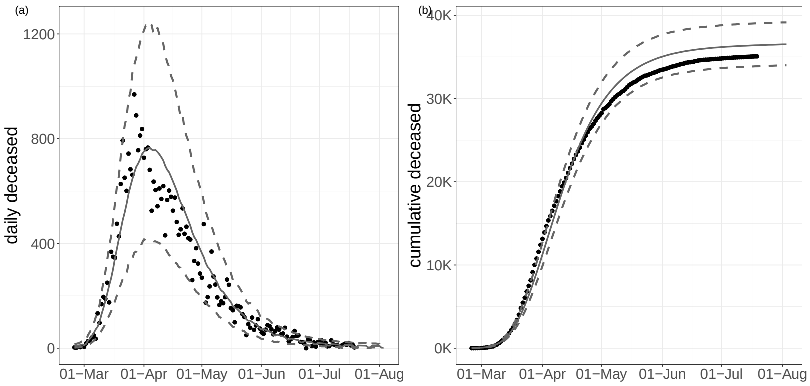

FIGURE 4 Observed (black dots) and fitted values (grey solid lines) with 95% confidence intervals (grey dashed lines) for model with baseline and week-day additive effect, estimated on the *daily deceased*.

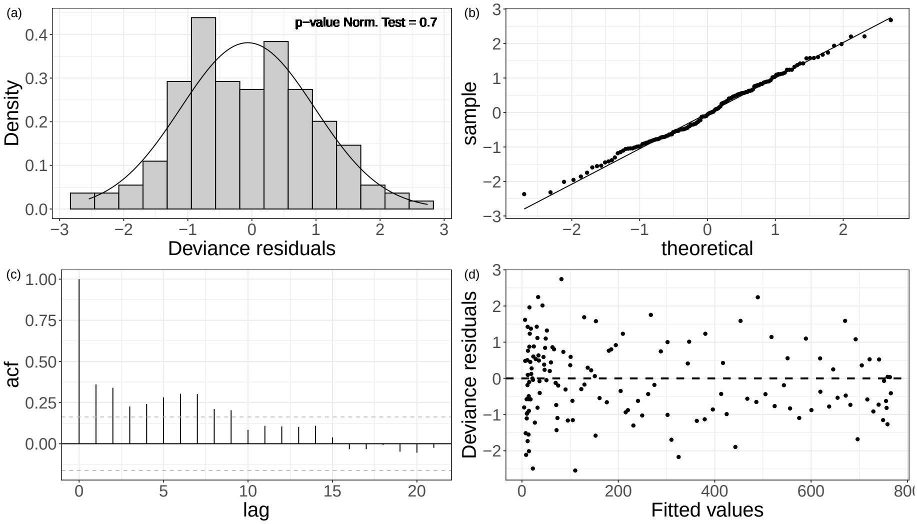

FIGURE 5 Deviance residuals for the model with baseline and week-day additive effect on *daily deceased*.

| Prediction of future cases and of the peak date

Validation performances on the *daily deceased* are analogous to the ones on *daily positives*. As in the main text, also here we highlight how the peak is accurately predicted with a shorter delay and generally smaller uncertainty for the *daily deceased* than for the *daily positives*. This is probably related to the more regular behavior of the series, due to a likely more homogeneous collection process of the records.

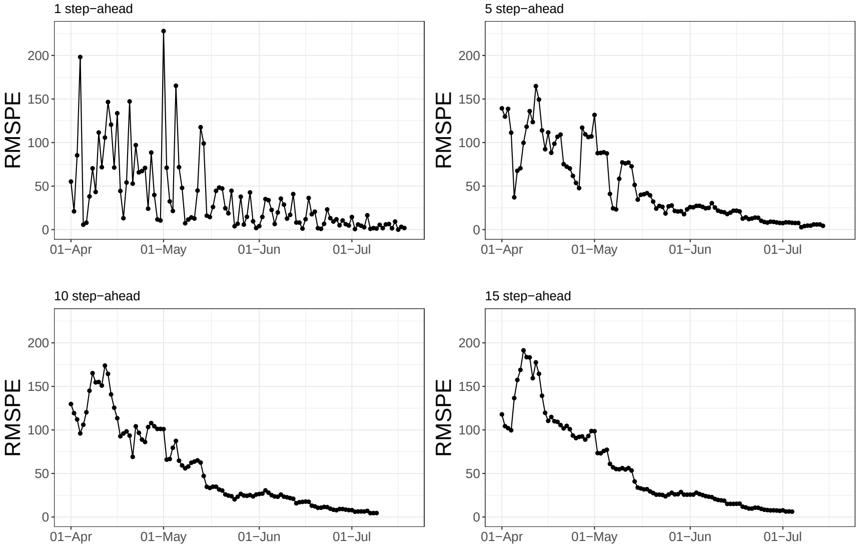

FIGURE 6 RMSPE for *daily deceased* at different steps-ahead.

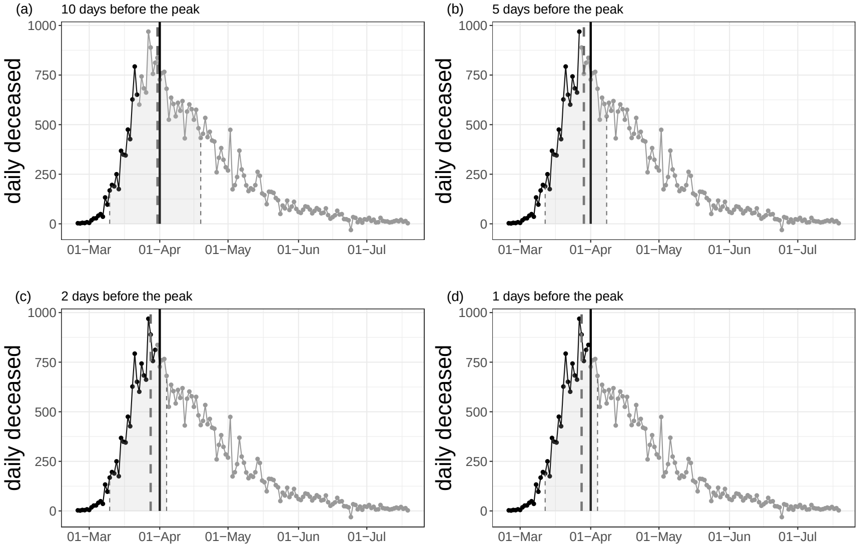

F I G U R E 7 Estimation of the date of the peak for *daily deceased* at different steps-before.

Regional *daily positives*

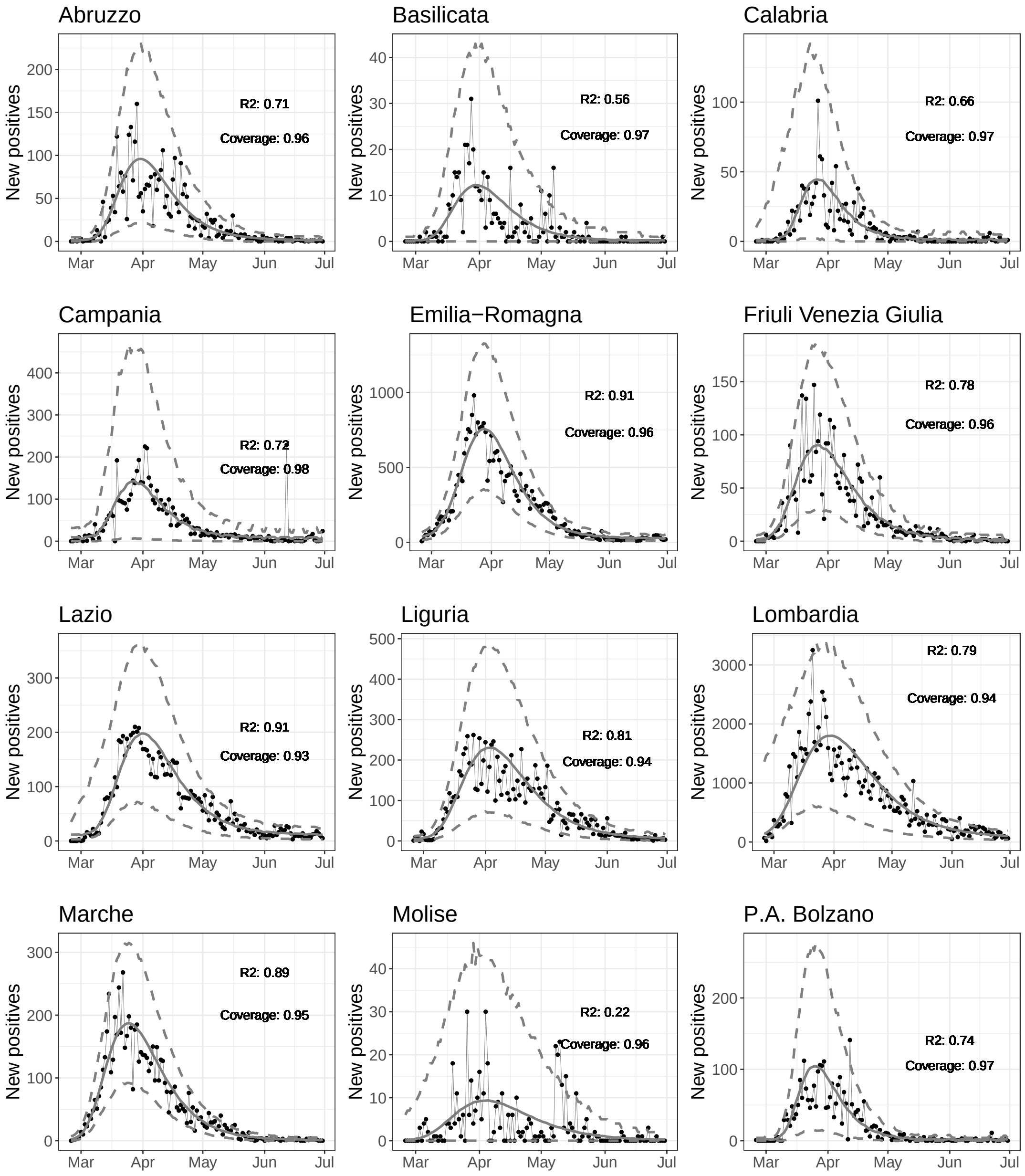

FIGURE 8 Observed (black dots) and fitted values (grey solid lines) with 95% confidence intervals (grey dashed lines) for model with baseline and week-day additive effect, estimated on the *daily positives* at the regional level.

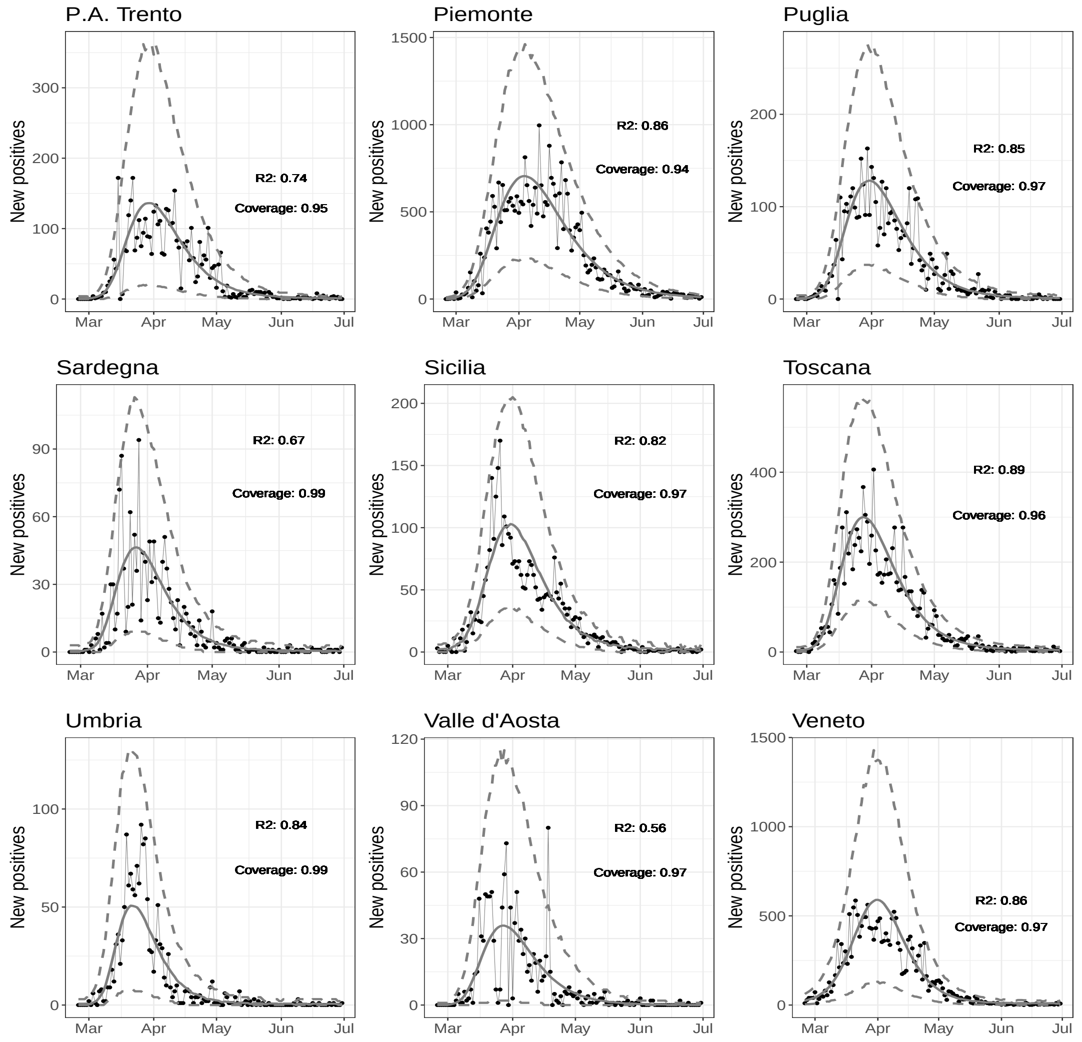

FIGURE 9 Observed (black dots) and fitted values (grey solid lines) with 95% confidence intervals (grey dashed lines) for model with baseline and week-day additive effect, estimated on the *daily positives* at the regional level.

Regional *daily deceased*

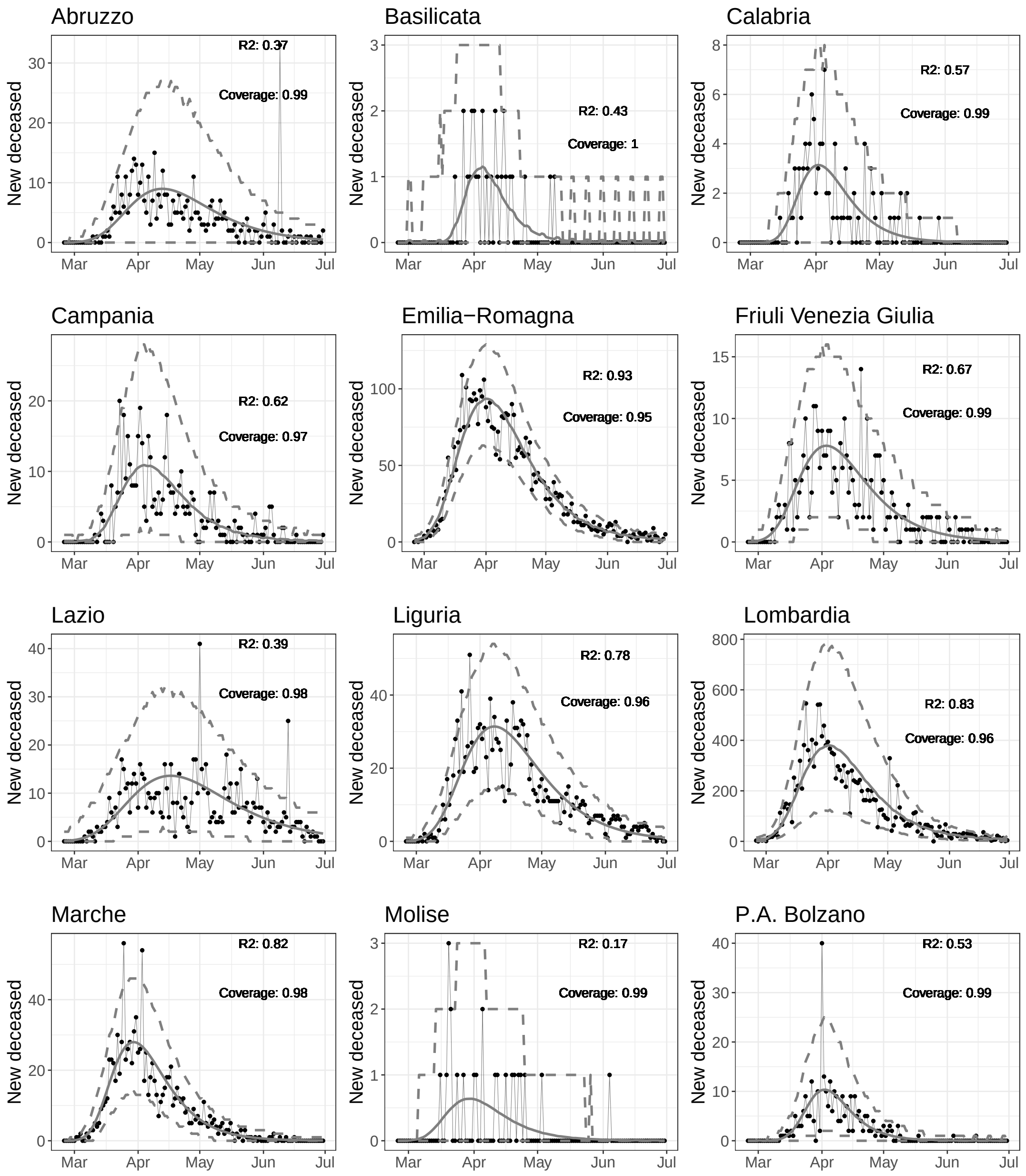

FIGURE 10 Observed (black dots) and fitted values (grey solid lines) with 95% confidence intervals (grey dashed lines) for model with baseline and week-day additive effect, estimated on the *daily deceased* at the regional level.

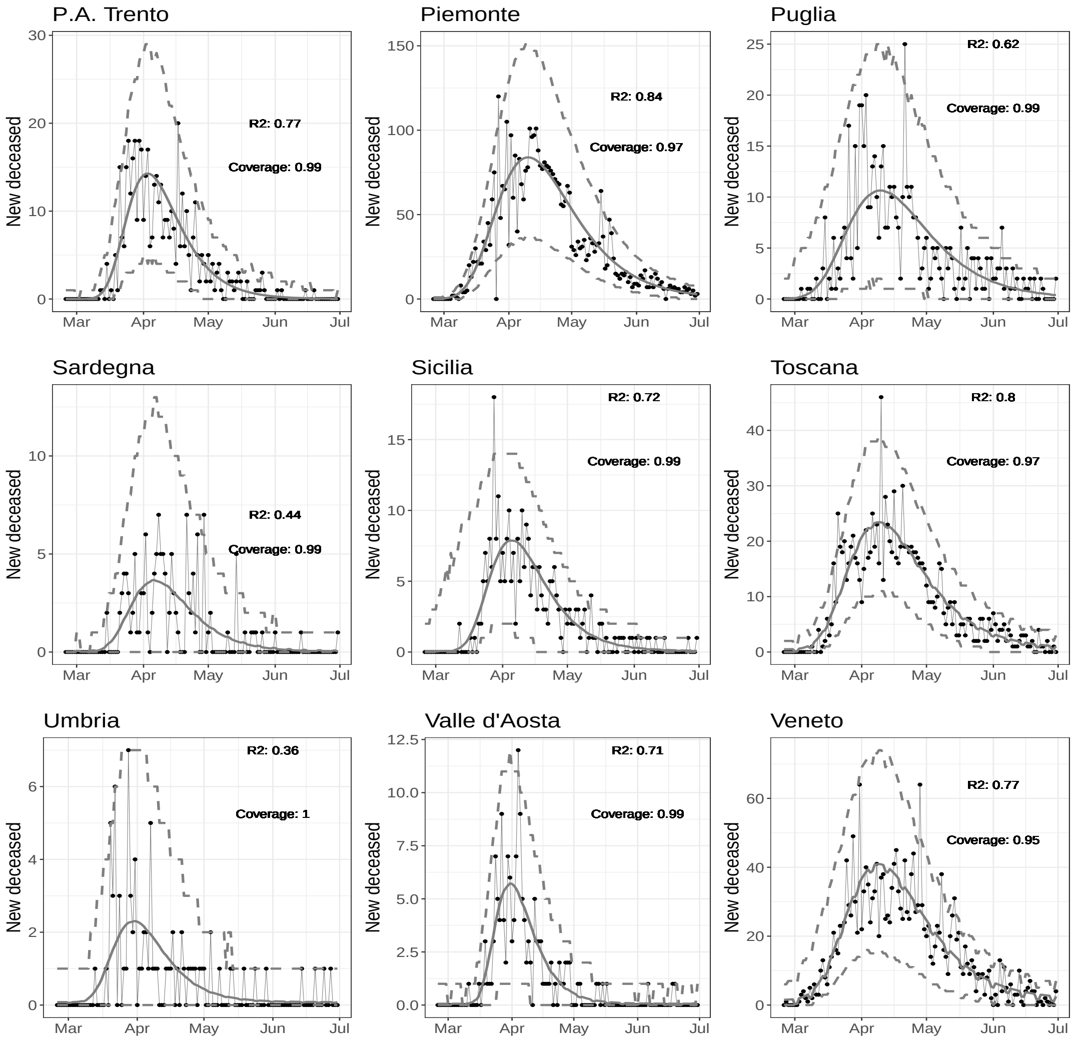

FIGURE 11 Observed (black dots) and fitted values (grey solid lines) with 95% confidence intervals (grey dashed lines) for model with baseline and week-day additive effect, estimated on the *daily deceased* at the regional level.
